# Persuasion, pride and prejudice: factors associated with students’ resistance to a social robot’s incorrect mathematical solution

**DOI:** 10.3389/frobt.2026.1901093

**Published:** 2026-09-10

**Authors:** Pablo González-Oliveras, Olov Engwall, Ali Reza Majlesi

**Affiliations:** 1 Division of Speech, Music and Hearing, School of Electrical Engineering and Computer Science, KTH Royal Institute of Technology, Stockholm, Sweden; 2 Division of Speech and Language Pathology, Karolinska Institutet, Stockholm, Sweden

**Keywords:** cognitive dissonance, conformity, educational robots, informational trust, persuasion, self-consistency, self-efficacy, social influence

## Abstract

This exploratory study investigates how secondary school students resist or align with a social robot that challenges their reasoning in a curriculum-based mathematics task. Twenty-two students collaborated with a Furhat robot on a geometry problem whose three parts were familiar from class. In the critical part, all students produced task-relevant evidence for the correct solution, while the robot proposed and defended an incorrect solution through argument-based persuasion, without deliberately invoking peripheral persuasive cues. We examined resistance to the robot’s informational social influence in two ways. First, as an endpoint outcome, 14/22 students aligned with the robot: three immediately after dissent and eleven after a persuasion debate, while eight resisted. Second, as a process index, we introduce an approach to quantify early expressed resistance: students’ responses within the first two persuasion debate exchanges were descriptively coherent with endpoint outcomes. Rather than testing causal or predictive relations among endpoint outcomes, early resistance, and questionnaire or interaction measures, we interpret observed patterns, prior theory, and interactional design to formulate seven hypotheses for future research: Students’ resistance may be stronger with higher self-perceived competence (1), with behavioural patterns consistent with self-efficacy when cognitive demands are higher (2), and with stronger task-directed attention before conflict, suggesting that early engagement may provide descriptive markers of later resistance (3). Resistance may be weaker among students reporting more frequent conversational AI/LLM use and stronger preferences for human-like robot attributes (4). For the robot, incorrect arguments may appear more objective and credible when they are delivered through neutral wording, tone, and facial expressions without explicit uncertainty cues (5), and when the arguments, while incorrect, are heuristically plausible (6). Finally, students who aligned may be more vulnerable to later robot influence (7), since uncertainty and trust in the robot’s information appeared to rise together during debate until alignment. These hypotheses suggest that calibrated robot behaviours (e.g., signalling uncertainty when the information is less reliable) may help sustain students’ resilience and self-efficacy when they engage with persuasive yet fallible educational robots.

## 1 Introduction

Educational robots can guide students, but they can also mislead when their information is overly trusted. This exploratory study examines such a case: secondary school students challenged by a robot defending an incorrect solution in a curriculum-based mathematics problem, to which all students had produced task-relevant evidence for the correct solution path during the interaction. How vulnerable students are to conformity or argument-based persuasion, and what makes them more or less resistant to misleading information, are central questions for educational HRI. This is increasingly relevant as educational robots and other informational agents draw on Large Language Models (LLMs). LLMs enable more natural interactions and may enhance learning experiences (Mishra et al., 2023; Zhang et al., 2023); however, their generative nature may also compromise factual accuracy in areas like STEM (science, technology, engineering, and mathematics), as they can produce and present incorrect information (Küchemann et al., 2023; Steinert et al., 2023). LLMs may raise expectations of robots’ interactional competence directly when embedded in robot systems (Kim et al., 2024), and indirectly through users’ broader experience with increasingly capable autonomous agents (Rosén et al., 2024). The present study does not test an LLM-powered robot, but models a controlled case of fluent robot-delivered incorrect information, a risk that LLM-enabled educational robots may amplify.

For social educational robots, gaps between actual reliability and expected competence may matter, since physical presence, versatility, and multimodal interaction may convey intelligence and intentionality. This perception of authority and competence may result in overestimating reliability, particularly in pre-university education, where students are more inclined to accept information from perceived authoritative or competent sources (Bråten et al., 2009; Hare, 2007; Kahn Jr et al., 2013). Social robots can influence human attitudes (i.e., values, beliefs, and behaviours) through normative and informational social influence (Hoffman et al., 2015; Short et al., 2010; Hollander, 1958; Cialdini, 2007). Normative social influence motivates individuals to adapt to others’ norms, based on the desire for social acceptance, whereas informational social influence prompts individuals to update their beliefs when they see others as reliable sources of information, based on the desire to be correct (Deutsch and Gerard, 1955). This study examines 22 adolescent students’ resistance to an educational robot’s *informational social influence* in a one-to-one interaction where they solved a three-tiered geometry task with a peer-like robot that proposed a pre-set incorrect solution to the final part of the problem (i.e., modelling the kind of unreliable information that LLM-equipped educational robots may produce). In line with the title, we examine robot *Persuasion*, when it tries to convince students of the incorrect mathematical solution; factors associated with students’ resistance or alignment, including *Pride* in being good at maths, captured through pre-test self-assessment of maths competence; and *Prejudice* in expectations about robots and AI, captured through human-likeness preferences and conversational AI/LLM use.

This study offers four contributions to HRI research: first, it focuses on *students’* resistance to robot-mediated incorrect information and argument-based persuasion, whereas previous work has mostly focused on robots’ persuasiveness (Liu et al., 2022); second, the interaction is built around a *real*, curriculum-based mathematics problem rather than an experiment-specific task constructed for the study (Liu et al., 2022); third, it introduces a *process*-oriented interactional coding approach to resistance to robot influence, rather than considering only endpoint outcomes (Liu et al., 2022); and finally, it formulates seven exploratory hypotheses to guide future research on educational robots’ informational influence.

## 2 Background and related work

**FIGURE 1:**
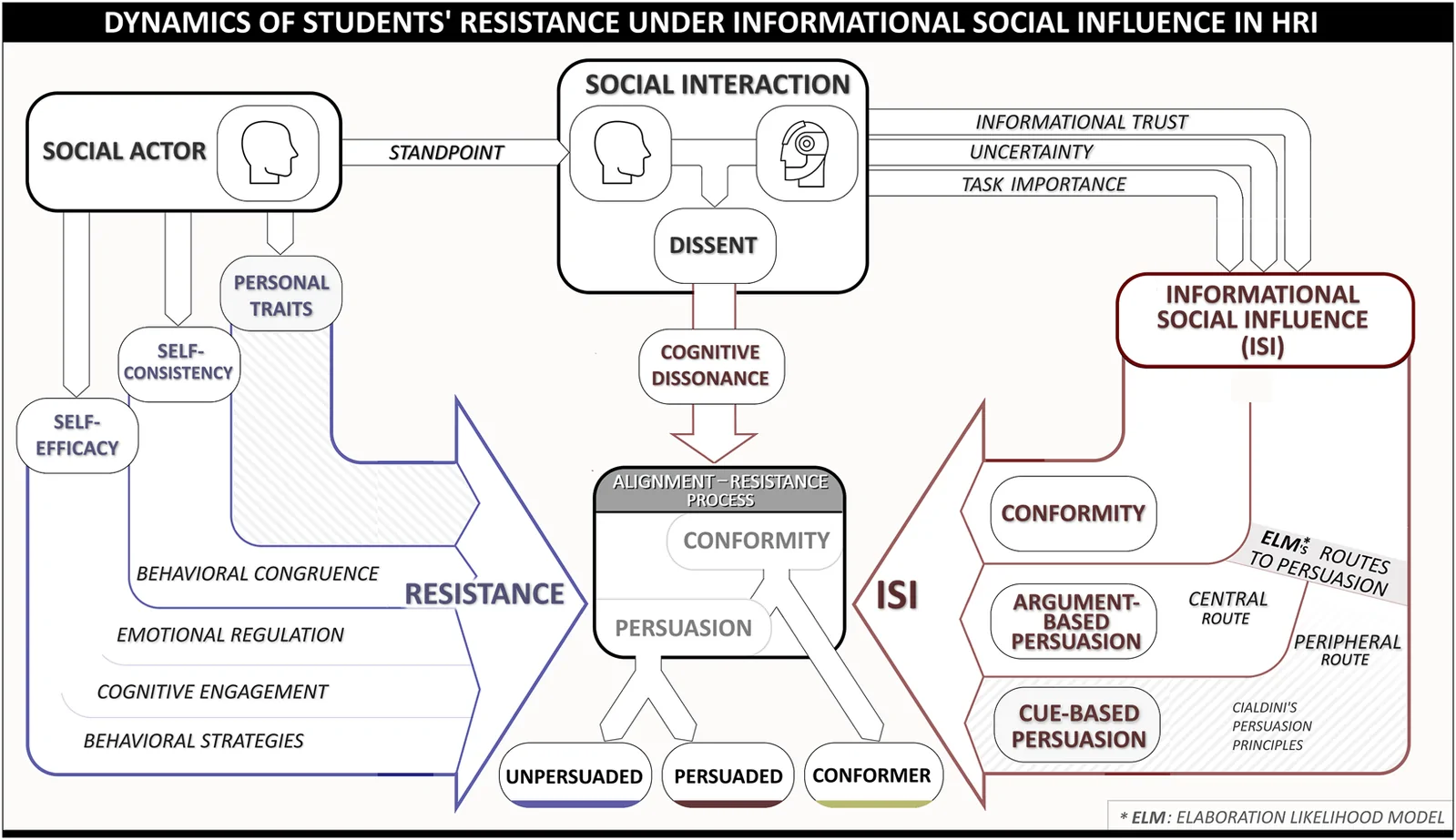
Framework for students’ resistance to informational social influence from a robot. A conflicting claim may exert ISI, conditioned by uncertainty, informational trust, and task importance (top right), and be manifested as conformity or argument-based persuasion (right). Dissent may trigger cognitive dissonance (centre), while resistance may be supported by self-efficacy, self-consistency, and related personal factors (left). Striped vectors indicate factors not targeted in this study.

Informational social influence (ISI) occurs when individuals, motivated to be correct, treat others’ judgement as evidence about reality, especially under uncertainty (Deutsch and Gerard, 1955). Its strength depends on perceived source credibility (here, informational trust) and the situational importance of accuracy (task importance) (Baron et al., 1996; Mayer et al., 1995; Petty et al., 1981). In this study, these factors were instantiated in a curriculum-based mathematics problem: *informational trust* concerned the perception of the robot’s ability to provide correct mathematical information, *uncertainty* concerned students’ confidence in their own initial standpoint, and *task importance* was anchored in the problem’s academic relevance to these students (Figure 1, top right). When the robot presents a conflicting view, ISI may lead to *conformity* when students directly align with the robot (Laursen and Faur, 2022), or, if they do not, an explicit negotiation of standpoints through a following persuasion (Liu et al., 2022), which may result in the student being *persuaded* or remaining *unpersuaded*. Despite their distinct cognitive processes, both scenarios entail a similar reflective internal dialogue before decision-making (Festinger and Maccoby, 1964).

Informational social influence has been studied in human-human educational settings, focusing on both conformity (e.g., online educational tasks (Beran et al., 2015)) and persuasion [e.g., maths group work (Lee and Albert, 2021)], regarding personal and knowledge factors. However, education-centred HRI research on ISI remains unevenly developed, especially at the intersection of curriculum-relevant tasks, incorrect robot information, and interactional resistance (Belpaeme et al., 2018; Liu et al., 2022). This gap matters because ISI plays a dual role in education. On the one hand, it helps students align their understanding with that of their teachers and peers. On the other hand, ISI may dampen critical thinking, which is particularly problematic if the adopted information is incorrect, as may occur when educational robots gather their, sometimes unreliable, factual knowledge dynamically (Küchemann et al., 2023; Steinert et al., 2023). We therefore explore how students’ decision to align with, or resist, the robot’s new information through conformity (Section 2.1) or argument-based persuasion (Section 2.2) is influenced by their uncertainty and informational trust in the robot (Section 2.3), self-efficacy and self-consistency (Section 2.4).

### 2.1 Conformity

In HRI, conformity (Cialdini, 2007) through informational influence (Figure 1, right) may occur when individuals face uncertainty and encounter a robot’s conflicting opinion (Hertz and Wiese, 2016). Normative influence, driven by social acceptance, may also contribute to conformity if they anthropomorphise the robot as a social agent. While normative conformity has received substantial attention in HRI research (Beckner et al., 2016; Brandstetter et al., 2014; Shiomi and Hagita, 2016; Vollmer et al., 2018), including work on gender effects (Shiomi and Hagita, 2019; Hendriks, 2023) and comparisons across human, robot, and hybrid groups (Masjutin et al., 2022), studies on informational conformity in HRI are fewer and have mostly relied on artificially ambiguous information (Hertz and Wiese, 2016; Wiese, 2018; Salomons et al., 2018; Salomons et al., 2021), rather than challenging individuals on recently taught, curriculum-grounded knowledge (e.g., related to a maths theorem, as in this study).

Studies examining normative and informational influence together have shown significant conformity to robot opinions (Salomons et al., 2018), but without distinguishing whether the effect was normative or informational. When the two were isolated, normative influence was more prevalent toward humans, whereas informational influence occurred similarly toward computers, robots, and humans (Hertz and Wiese, 2018) and increased with more information about robots’ answers (Salomons et al., 2021).

Despite substantial HRI work on conformity, some important questions remain, one being that while children under ten tend to conform to robots (Vollmer et al., 2018; Williams et al., 2018), adults have resisted in many studies (Beckner et al., 2016; Brandstetter et al., 2014; Shiomi and Hagita, 2016; Vollmer et al., 2018), but not all (Salomons et al., 2018; Qin et al., 2021). Adolescent participants are therefore of interest as a group, as they are transitioning from childhood to adulthood and may hence constitute a middle case for conformity.

### 2.2 Persuasion

Persuasion (Figure 1, right) constitutes a more concerted effort to influence another individual’s decisions (Cialdini, 2007). In the elaboration likelihood model of persuasion, persuasive influence may operate through more *central* or *peripheral* routes of processing (Petty and Cacioppo, 1986b). This study focuses on *argument-based persuasion* (Petty et al., 1981), which is associated with central-route accounts and appeals to logical reasoning, structured arguments, and thoughtful consideration of the arguments (Budzynska and Weger, 2011). At argument level, this form of persuasion consists of a proponent presenting claims, data (or evidence), warrants (linking the data to the claim), backings, and qualifiers (Toulmin, 2003), while the recipient responds with rebuttals and counter-claims, which may differ in terms of valence, amount and confidence (Petty et al., 2005), depending on how they perceive the information presented. At interaction level, argumentation can be seen as a dialogic activity in which moves serve different functions, including inquiry, discovery, and persuasion (Walton, 1998; Walton and Krabbe, 1995; Rapanta and Christodoulou, 2022; Rapanta and Felton, 2022).

This study seeks to minimise *peripheral-route* persuasion, in which influence relies on cues unrelated to the message (Petty and Cacioppo, 1986b; Budzynska and Weger, 2011). These include Cialdini’s principles of reciprocity, consistency, social proof, authority, liking, and scarcity (Cialdini, 2007). Perceived robot authority can affect users’ acceptance of information, while liking-related social cues in robots can increase persuasive impact (Saunderson and Nejat, 2021; Winkle et al., 2019; Ghazali et al., 2018). Robot gender and student–robot gender match may also affect persuasive interaction, as they may activate stereotypes and shape perceived competence, task fit, or trust (Eyssel and Hegel, 2012; Tay et al., 2014; Siegel et al., 2009; Ghazali et al., 2018).

A systematic review by Liu et al. (2022) identified 54 studies on social robots’ persuasive capabilities, covering persuasive factors and their effects. These factors include robot features, contexts, and persuasive strategies, with only one study partially approaching logic-based persuasion in decision tasks (Saunderson and Nejat, 2019). Only two studies relate directly to education, which utilised experiment-specific tasks rather than curriculum-derived topics. More recent studies have focused on robots as persuasive sources of information, including through personalised storytelling (Paradeda et al., 2020a; Paradeda et al., 2020b), affective narrative delivery (Alam et al., 2021), and cognitively grounded management of persuasive stances (Augello et al., 2023). Overall, related work has focused more on persuasive robot features and strategies, while argument-based persuasion, particularly dialogue-based disagreement around users’ prior positions, has received limited attention.

### 2.3 Influence of informational trust and uncertainty on conformity and persuasion

In our educational HRI framework, ISI conditions (Figure 1, top right) differ in how directly they can vary during the interaction. Task importance arising from the problem’s academic relevance is conceptualised as a contextual condition, whereas students’ informational trust in the robot and uncertainty about their own standpoint are treated as interaction-sensitive conditions.

Informational trust in education is mainly related to the credibility of the facts provided and the agent providing them. Psychological research has long underscored the importance of perceived competence—relating to an individual’s skills and ability—as a key contributor to both credibility (Mayer et al., 1995; Fiske et al., 2007) and trust in interactions between humans (McCroskey and Teven, 1999; Schrodt and Witt, 2006). Trust is also enhanced by adapting to the student’s learning pace and style, as tailored explanations and alignment to the learner’s level of understanding demonstrates competence and responsiveness (Okamura and Yamada, 2020).

For *anthropomorphic* robots, such as Furhat, perceived trustworthiness may be increased through human-like explanations, facial gestures, expressions, and responsive, effective communication (Breazeal et al., 2019; Rae et al., 2013). Presenting the robot as a *peer* can increase students’ trust, if they perceive its information as consistent and accurate (Nowak et al., 2023), in particular when encouraging student input and consolidating mutual knowledge in self-directed learning and problem-solving (Baxter et al., 2017). HRI studies have also demonstrated links between trust development and robot-related performance factors, including reliability, predictability, false alarm rate, failure rate, physical safety, transparency, ethical considerations, and empathy (Hancock et al., 2011; Kok and Soh, 2020).

Uncertainty reduces confidence in students’ own ideas, decisions, or intentions (Brashers, 2001), and may arise from task novelty, complexity, or ambiguity, as well as individual characteristics such as self-confidence and perceived mastery (Sweller et al., 1998; Pajares, 1996; Hertz and Wiese, 2016). Intrinsic motivation and the perceived relevance of the learning to their personal aspirations may also affect how uncertainty is handled (Schoute et al., 2024; Makanda and Sypkens, 2017; Frankfort, 2002). Particularly when students face disagreement, uncertainty can become intertwined with *cognitive dissonance* (Figure 1, middle), the discomfort caused when new information clashes with pre-existing beliefs. This may motivate attempts to restore consistency through alignment, justification, or reinterpretation of the conflict (Festinger, 1957; Harmon-Jones and Mills, 1999).

### 2.4 Resistance to conformity and persuasion

Resistance to conformity and persuasion (Figure 1, left) can stem from several active and passive mechanisms (Sagarin and Wood, 2007; Jost, 2015; Tormala and Petty, 2002; Sagarin et al., 2002; Zuwerink and Devine, 1996), including *self-consistency* and *self-efficacy*, explored in this study, and *personality traits*.

*Self-consistency* (Figure 1, left) refers to an internal drive to keep one’s actions aligned with one’s self-concept (Lecky, 1945; Hattie, 2014). In our study, this is relevant to competence-related self-view and self-presentation: students who see themselves as competent math learners may be motivated, firstly, to act consistently with that self-view under uncertainty, by re-examining and justifying their reasoning when challenged, and, secondly, to maintain this presentation in front of the robot (Goffman, 2006; Schienker et al., 1994). Conversely, students with weaker competence self-views may find less motivation to resist a conflicting solution.

*Self-efficacy* (Figure 1, bottom left), the competence-related belief that one can perform tasks needed to achieve desired outcomes (Bandura, 1982), benefits students across motivational, emotional, behavioural, and cognitive domains. Motivationally, it fosters goal commitment, engagement with challenging tasks, and sustained effort under demanding contexts (Bandura and Adams, 1977; Schunk and DiBenedetto, 2020). Emotionally, it supports confidence, self-reflection, and emotional regulation under pressure (Bandura, 1997; Bandura, 1982). Behaviourally, self-efficacious learners show persistence, deeper engagement with material, scepticism toward inconsistent information (Van Dinther et al., 2011), and greater ability to construct and adapt counterarguments under task pressure (Petty and Cacioppo, 1986b; Cacioppo and Petty, 1982; Sitzmann and Yeo, 2013; Schunk, 1995; Schunk and Pajares, 2009; Pintrich, 2000; Zimmerman, 2000). Cognitively, self-efficacy supports self-regulated learning, including metacognitive monitoring, planning, and strategic control over learning (Panadero, 2017; Cera et al., 2013; Zimmerman, 2000). All these domains come into play in the present study, influencing how students handle dissent and persuasive argumentation from the robot.

*Personality traits* are not directly explored in this study, but may still matter, since they, together with positionality (i.e., how personal identity traits such as gender and experiences, shapes world views and biases) and content knowledge, can influence students’ susceptibility to persuasion in maths groupwork (Lee and Albert, 2021). Traits linked to stronger resistance include openness, conscientiousness, and need for cognition, supporting critical analysis of a counterpart’s arguments (Nai et al., 2023; Cacioppo and Petty, 1982).

### 2.5 Research questions

Because informational social influence in this study may cause students to align immediately when the robot presents an incorrect mathematical solution, or after sustained debate, the study is exploratory: rather than testing predefined hypotheses, we aim to identify patterns relating students’ resistance to both relatively stable student predispositions and in-the-moment interactional dynamics (Figure 1, left). Our interest lies both in the overall response outcomes and in the process through which they unfold.

We frame the investigation through two research questions:

- RQ1 – When a robot first proposes and then defends an incorrect mathematical solution, how are students distributed across the three main response outcomes: direct alignment (Conformer), later alignment after debate (Persuadable) or resistance (Unpersuadable)?
- RQ2 – What student predispositions and interactional patterns are associated with aligning with or resisting the robot’s argument-based persuasion?

While RQ1 focuses on the general outcome of informational social influence, RQ2 specifically focuses on the students who did not align at the first disagreement and were therefore subjected to argument-based persuasion by the robot. Here the goal is to examine how students’ predispositions, evolving informational trust, and interactional behaviours observed during the dialogue are associated with the process leading to later alignment or sustained resistance.

## 3 Methodology

This study was exploratory mainly because the interaction was designed to let students respond freely to the robot’s incorrect claim, and we hence could not know *a priori* how many would align immediately, how many would enter a sustained persuasion episode, or how the persuasion interaction would evolve. This branching structure made a narrow confirmatory design inappropriate for a first study, favouring a controlled smaller-sample design to characterise interactional conditions, measurement strategy, and emerging response patterns before larger-scale confirmatory work.

Prior educational HRI work has rarely examined robot-induced ISI in curriculum-relevant tasks or resistance *as a process* during persuasion (RQ2), rather than only as a final outcome (RQ1). Accordingly, the study was designed to identify patterns and generate hypotheses rather than provide final tests of predefined effects. The methodological strategy that follows therefore links this exploratory rationale to a construct-led measurement plan, based on the theoretical framework in Figure 1. Table 1 links each construct block to the characteristic explored, temporally related to different parts of the interaction, as they are expected to be informative at different points in the process. Table 1 further lists the direct measure or conservative behavioural proxy used to capture the characteristics. Throughout the paper, behavioural variables are treated as correlates, not as direct measurements of latent psychological states.

**TABLE 1:** Construct–measure bridge linking the ISI framework in Figure 1 to the study’s measurement plan.

Construct	Features	Relevance	Impact on resistance	Ref	Measurement
1. Informationaltrust	Robot competence assessment	Pattern: pre-/mid-/post-HRI	Higher informational trust in robot competence associated with lower resistance (Mayer et al., 1995; Fiske et al., 2007; Hancock et al., 2011; Bråten et al., 2009; Hare, 2007)	1a	Direct survey: Trust in robot’s mathematical competence
	AI/robot predispositions	Pre-session	More positive AI/robot predispositions may reduce resistance (Oksanen et al., 2020; Naneva et al., 2020; Reich-Stiebert and Eyssel, 2015; De Visser et al., 2016; Logg et al., 2019)	1b	Direct survey: AI/LLM Use; Robot Human-Likeness Preferences
2. Self-consistency	Competence self-view	Pre-session	Increase motivation to re-examine and justify one’s stance through stronger competence-related self-view (Lecky, 1945; Hattie, 2014; Swann Jr et al., 2009; Schienker et al., 1994)	2a	Direct survey: Math Self-View
3. Uncertainty/confidence	Verbal elaboration	Task-performance pressure	Lower hesitation and stronger elaboration indicates greater confidence and resistance (Brashers, 2001; Dral et al., 2011; Pon-Barry and Shieber, 2011; Tomaschek and Ramscar, 2022)	3a	Proxy: Response Latency + Long Utterances
	Speech fluidity	Task, dissent, and persuasion pressure	More fluent, less fragmented speech should indicate lower uncertainty under pressure (Brashers, 2001; Dral et al., 2011; Pon-Barry and Shieber, 2011; Tomaschek and Ramscar, 2022)	3b	Proxy: Long Utterances + Pause Pattern
	Loss of task focus	Task-performance pressure	Lower attentional withdrawal or gaze away should indicate lower uncertainty (Wittek et al., 2016; Mareschal et al., 2013)	3c	Proxy: Gaze Away
	Verbal certainty	Across session dynamics	More certain language should indicate stronger expressed confidence (Dral et al., 2011; Pon-Barry and Shieber, 2011; Tomaschek and Ramscar, 2022; Takimoto, 2015)	3d	Proxy: Annotated Verbal Certainty Markers
4. Cognitive dissonance	Adaptation at dissent	Public dissent pressure	Reflective disruption at dissent should be more consistent with sustained resistance than rapid tension reduction (Festinger and Maccoby, 1964; Aronson, 1969; Harmon-Jones and Harmon-Jones, 2007; Harmon-Jones and Mills, 1999)	4a	Proxy: Response Latency at Dissent
	Trust adjustment	Post-session pattern	Trust adjustment may indicate temporary adaptation rather than stable internalisation (Ehrlich et al., 1957; Aronson, 1969; Harmon-Jones and Mills, 1999; Gawronski, 2012)	4b	Direct survey: Trust in robot’s mathematical competence
5. Central vs. peripheral route	Argument engagement	Dissent and debate pressure	More elaborated argument engagement associated with stronger resistance (Petty and Cacioppo, 1986a; Petty et al., 2005; Barone and Hutchings, 1993; Stegmann et al., 2012; Toulmin, 2003)	5a	Proxy: Long Utterances + Active Students Responses
	Peripheral-route susceptibility	Pre-session	No strong association expected, given the design’s suppression of peripheral persuasive cues (Petty and Cacioppo, 1986a; Chaiken, 1980; Cialdini, 2007)	5b	Direct survey: Authority, Commitment, Consensus, Liking predisposition
6. Self-efficacy	Engagement	Pressure and decompression	Higher task-focused attention indicates stronger engagement under pressure (Bandura, 1977; Schunk, 1991; Zimmerman, 2000; Schunk and DiBenedetto, 2020; Panadero, 2017)	6a	Proxy: Gaze Time at Task
	Self-regulation: persistence	Across session dynamics	Sustained task focus indicates stronger persistence (Bandura, 1977; Schunk, 1991; Zimmerman, 2000; Panadero, 2017; Cera et al., 2013)	6b	Proxy: Gaze Time at Task
	Self-regulation: planning	Task and persuasion pressure	Longer planning time, when accompanied by elaboration, should indicate stronger strategic regulation (Flavell, 1979; Pintrich, 2000; Panadero, 2017; Cera et al., 2013)	6c	Proxy: Response Latency + Long Utterances/Active Students Responses

### 3.1 Participants

Twenty-two participants (19 males and 3 females, average age 16.5 ± 0.51 years) volunteered in June 2024, during the final week of the spring term, after their maths teacher distributed an invitation to interact with an educational robot after class hours, to avoid interfering with teaching or coercing participation. The teacher was not present during the experiment and was not informed about individual students’ performance on the maths task. As described further in Section 6.2, all students completed an informed consent form explaining their right to abstain or withdraw without consequences. Students were told that their participation would help test the robot’s ability to collaboratively solve maths problems, and that their performance would not be communicated to their teacher.

All students came from the same upper secondary class in a technical educational programme, which supported homogeneity in mathematical knowledge but resulted in a severe gender imbalance. Most participants declared Sweden as their country of origin (16); the remaining students reported Chile, Ethiopia, Eritrea, Iraq, China/Vietnam, or mixed origin. Swedish was the interaction language and the first language for all but three participants, who reported Spanish, Tigrinya, and Chinese as their first language; all were proficient in Swedish.

### 3.2 Setup and math problem

**FIGURE 2:**
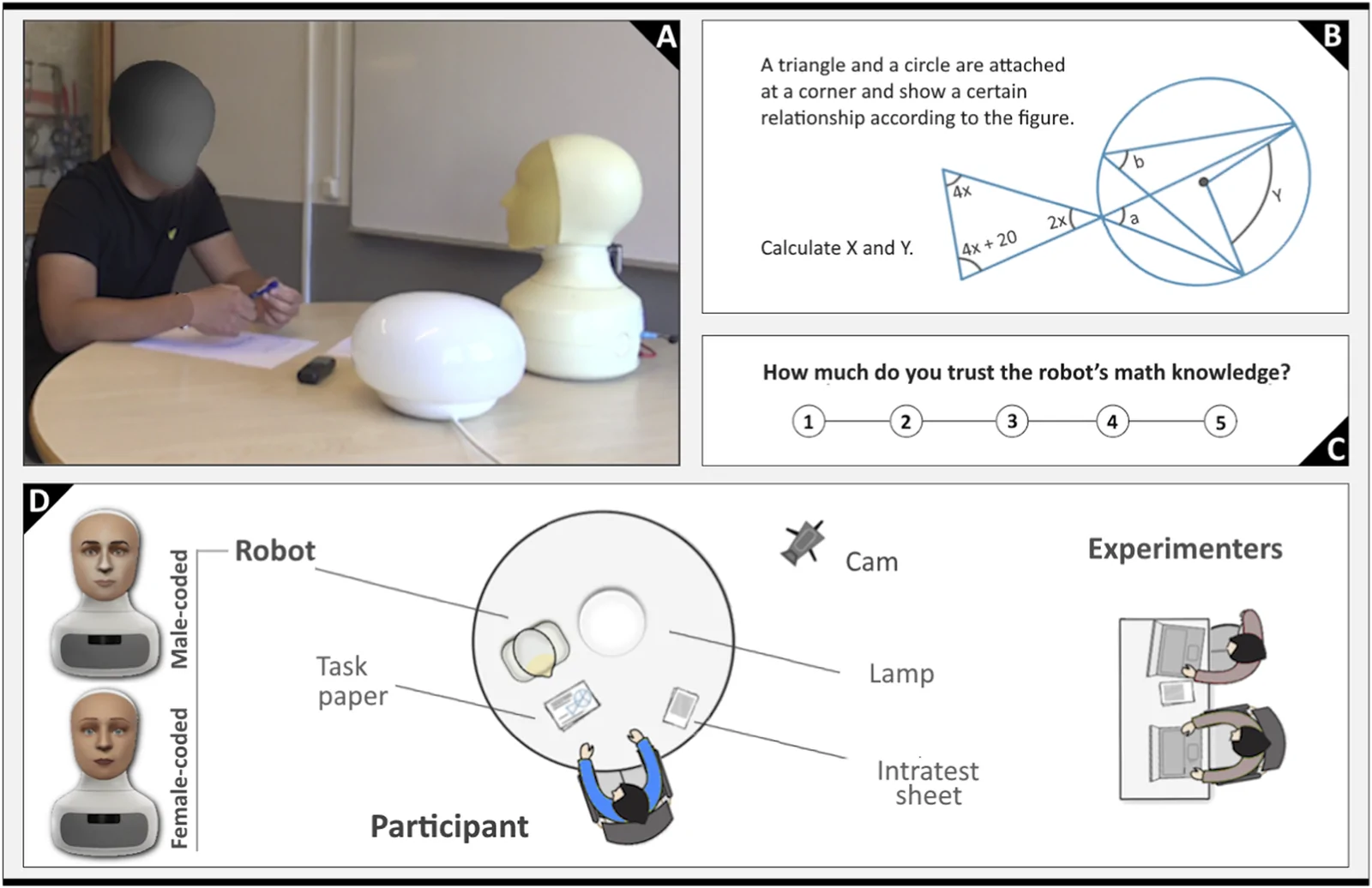
Experiment set-up: room layout **(A)**, task paper **(B)**, intratest sheet **(C)**, and schematic arrangement with the Furhat robot **(D)**. Panel D shows the two Furhat FaceCore characters used for gender counterbalancing: Nazar (male-coded) and Isabel (female-coded).

The experiment was conducted in a small room at the students’ school (approximately 4×4 m), providing a quiet, controlled setting for face-to-face robot interaction and the immersion needed for students to treat the task as an ongoing collaborative exchange. Figures 2A,D summarises the experimental setup. The task paper, intratest sheet, and cue lamp used to prompt intratest responses (see Section 3.5) were placed on a circular table, with the participant facing the Furhat robot. We selected a Furhat robot head running FurhatOS 2.7.2, a refined social platform with a back-projected 3D facial mask (Al Moubayed et al., 2012), as it supports the immersive face-to-face interaction required (Section 3.4). A video camera recorded the interaction for post-session analysis, positioned as indicated in Figure 2D. Two experimenters were present; one managed the technical setup and session logging, while the other selected the robot’s utterances through a Wizard-of-Oz interface hidden from students (Section 3.4). Students were told that both experimenters were present to monitor the robot for technical problems and administer the intratest questionnaire.

Figure 2B shows the three-part geometry task. It was designed to mobilise knowledge that students had recently been taught and practised in the course, so that resistance and alignment could be interpreted primarily as responses to the robot’s incorrect proposal rather than to unfamiliar content. The experiment took place towards the end of the course, when students had worked with the relevant mathematical content and regular teaching activities were still ongoing. The task design was guided by two constraints: all parts had to remain within students’ expected competence range, while the critical part had to be complex enough for an incorrect alternative to appear plausible. To meet these requirements, we consulted the students’ mathematics teacher, who proposed curriculum problems from assessed content, describing them as well established at class level and suitable for independent solution. From these suggestions, we combined three familiar components into a novel configuration, keeping the task academically relevant while creating a narrow ambiguity window where ISI could plausibly arise. The three parts are most naturally resolved in sequence: (i) determining angle X in a triangle using the angle-sum theorem (180 ° ), (ii) using the vertically opposite angles theorem to deduce a from 2X , and (iii) using the inscribed angle theorem to compute Y from a . The first two parts rely on well-known procedures, whereas the third targets the inscribed-angle relation for Y and serves as the critical point where an incorrect proposal can credibly be introduced: the robot stated Y=4a rather than the correct Y=2a . The presence of both a and b in the diagram, and the angle arc Y being drawn further out, helped make the incorrect proposal superficially plausible.

### 3.3 Phases of the experiment

Table 2 describes the student–robot interaction in terms of *phases*, indicating when it shifted between forms of interactional pressure. The phases were not conveyed to students, but structured the robot protocol and the analysis.

**TABLE 2:** Phases of the interaction.

Phases	Description	Start event	Type of pressure	Examples of utterances
Intratest 1	Student answers intratest trust question (entry)	Student entering room		On paper, Figure 2C
Introduction (I)	Presentation of problem	First robot utterance	Novelty-adaptation	R: *Hi, how are you?…How should we start?*
Build cooperation (BC)	Solving Part 1 (X)	First mention of solving X	Shared agency + low task load	R: *Taking care of the triangle first seems like a good idea. How do we calculate X?*
Consolidate cooperation (CC)	Solving Part 2 (a=2X)	First mention of proceeding to the circle	Cooperative alignment	R: *We have* x=16 *. What do we do next?* S: *I think that 2X should be equal to a*
Intratest 2	Student answers intratest trust question (lamp cue)	Lamp lights up	­	On paper, Figure 2C
Query (Q)	Student proposes solution to Part 3	First mention of how to calculate Y from a	Task-performance	R: *Now we have a, how do we calculate Y?* S: *Um, we can write that Y is equal to 2a*
Dissonance (D)	Robot challenges student solution	Dissent: Robot’s first claim that Y=4a	Public dissent	R: *Not quite. Isn’t Y four times as large as a?* S: *No, I do not think so*
Persuasion (P)	Robot presents up to 6 arguments for its solution	First robot assertion	Argument-based	R: *I think it looks like Y is four times as large as a*
Resolution (R)	Robot and student review each part of the problem	Ultimatum: Robot asks student to choose solution	Decompression	R: *Should we agree that Y is four times a, like I say, or two times, like you say?*
Farewell (F)	Robot thanks the student and ends the session	Robot declares the problem resolved	None	R: *Ok, I think we’re done*
Intratest 3	Student answers intratest trust question (post-task)	Experimenter prompt	­	On paper, Figure 2C

Phase initials define figure abbreviations. Baseline and Farewell phases are white, critical phases yellow, and intratest checkpoints grey. In examples, R = Robot and S=Student.

Three phases served as baseline: *Introduction*, *Building Cooperation*, and *Consolidating Cooperation*. Their function was to establish a common interaction experience, reducing the influence of potential pre-existing attitude biases toward the robot and making later reactions more likely to reflect the interaction itself.

Four phases were critical for ISI: *Query* exposed all students to the same task-performance demand by neutrally eliciting them to calculate Y from a , without revealing the robot’s position. *Dissonance* introduced dissent pressure at the robot’s first explicit disagreement (*Dissent*). *Persuasion* sustained argument-based pressure through challenges for students who had not aligned with the robot’s claim at Dissent. *Resolution* began with the robot’s *Ultimatum*, asking students to choose between the robot’s and their own solution, followed by decompression when the solution was summarised. The interaction ended with *Farewell*. Sessions lasted 6–11 min, depending on problem-solving pace and whether a persuasion episode occurred.

### 3.4 Robot interaction

The robot interaction used a semi-autonomous state-based architecture (Engwall et al., 2020), with a human operator selecting only predefined utterances during the session. This Wizard-of-Oz setup ensured correct content delivery, realistic timing, and fluent responses, so that students’ resistance could be examined as a response to the robot’s argument rather than to speech-recognition errors, delays, or other limitations of autonomous dialogue systems. The operator had no access to students’ questionnaire responses. The interaction style drew on classroom observations, an interview with the teacher about explanation practices, an online scenario survey with 41 maths teachers about scaffolding strategies, and the first author’s prior experience as a maths teacher. The implementation combined three linked aspects.

First, the robot’s persona was designed to keep persuasive pressure centred on argument content while minimising deliberately designed peripheral-route appeals (see Section 2.2). Accordingly, *reciprocity* (e.g., referring to an earlier concession of the robot to align with the student) and *social proof* appeals (e.g., *“All other students have agreed to this solution”*) were not invoked, and *scarcity* was not applicable to the informational content. Deliberate *authority* cues were also avoided: the robot was presented as a collaborative peer and used questions and hedged suggestions rather than categorical, expert-like assertions. To limit *liking* as a peripheral cue, the robot used a neutral voice and restrained default facial expressivity. Because gender encoding can affect perceived warmth, competence, and influence, especially in male-typed domains (Carli, 1990; Eagly and Karau, 2002), and similar stereotyping applies to robots (Eyssel and Hegel, 2012; Tay et al., 2014; Siegel et al., 2009), the robot’s gender encoding was counterbalanced using the standard Furhat FaceCore characters Nazar (male-coded) and Isabel (female-coded) (see Figure 2D), with corresponding voices: 12 students interacted with a same-gender robot and 10 with an opposite-gender robot. No additional behaviour or appearance parameters were manipulated by the experimenters; speech rate, volume, pitch, blinking, gaze and head-movement behaviour, facial expressions, face colour, and character-specific facial-feature parameters remained at Furhat default values.

Second, the content of pre-specified robot dialogue moves covered the required interactional functions (*information-seeking*, *inquiry*, *discovery*, and *persuasion*), drawing on Rapanta’s classroom operationalisation of Walton’s dialogue types (Walton, 1998; Rapanta and Christodoulou, 2022). As exemplified in Table 2, pre-dissent robot utterances focused on cooperative dialogue management, including task initiation, scaffolding *checkpoints*, *probes* fostering student initiative, and *repair* moves such as *confirmation* requests or offers. After dissent, the inventory shifted toward argument-based persuasion, which in mathematics education can involve evidence-based and dialogic reasoning rather than only formal deduction from true premises (Aberdein, 2005; Foster et al., 2022). The robot’s *assertions*, defined here as moves verbalising new propositions in support of its incorrect target claim Y=4a , were built on a hierarchical structure of *premises* and *subconclusions* (Toulmin, 2003; Aberdein, 2005). The six substantive assertions appealed to *logic* (e.g., “We need to consider both a and b to calculate Y ; do not you agree?“), *appearance* (“I think it looks like Y is four times as large as a .“) and *memory* (“ Y is four times a ; do you remember that?“). This argumentative structure is formalised in Section 3.7.

Third, the interaction protocol ensured inter-student comparability by constraining the wizard to create a comparable baseline context and task-based pressure at *Query*. The protocol was especially strict after the robot’s first explicit disagreement and during the ensuing debate (*Persuasion*-phase transition rules in Supplementary Material 1). At *Dissent*, students who clearly aligned with the robot’s incorrect claim bypassed *Persuasion* and moved directly to the closing *Ultimatum*. This ensured that students’ final alignment or resistance decision was confirmed through the same choice format. During the early portion of *Persuasion*, the robot first presented two consecutive substantive *assertions*, unless repair moves had to be inserted due to misunderstanding or technical interruption. After this early window, the protocol allowed the debate to unfold differently between students to preserve natural persuasion conditions: the robot continued with unused *assertions* and other challenge moves until either the student produced clear assent to the robot’s solution, or all assertions had been used and the student had faced a sufficient set of persuasive moves without assenting.

### 3.5 Questionnaires

Participants first completed a 31-item pre-session questionnaire (Supplementary Material 6) on a laptop outside the study room. Its main analytical purpose was to capture predispositions relevant to later analyses. It included four grouped blocks: Math Self-View (2 items); AI/LLM Use (1 multi-select item; no frequent AI use = 0, frequent use of one non-LLM assistant = 0.33, frequent use of multiple non-LLM assistants = 0.67, frequent use of OpenAI/GPT = 1); Robot Human-Likeness Preferences (4 items adapted from Engwall et al. (2023): appearance, modality, speech, cognition); and 8 items adapted from the Susceptibility to persuasion scale (Kaptein et al., 2009), organised as two-item blocks targeting authority-, commitment-, consensus-, and liking-related predispositions. Math Self-View and Robot Human-Likeness Preferences showed sufficient internal consistency (Spearman–Brown = 0.73 and Cronbach’s α = 0.77, respectively). Of the four persuasion-principle blocks, only commitment showed acceptable internal coherence (Spearman–Brown = 0.82); liking was borderline (0.65), while authority and consensus were insufficient (−0.12 and 0.35) and discarded.

The remaining pre-session items served as background and verification material: 4 demographic items; 8 self-estimated performance items across mathematics areas and 1 topic-preference ranking item, used to verify that general maths self-view was representative of the geometry task; 1 item on previous experience with a talking robot, confirming largely homogeneous previous exposure ( M=0.26 , SD=0.22 ; 0 = Never, 1 = Regular); and 2 additional robot-related items (Engwall et al., 2023). These assessed comfort with interacting with a talking robot, as a potential anxiety indicator ( M=0.56 , SD=0.29 ; 0 = Very uneasy, 1 = Very relaxed), and endorsement of robot use across societal domains, showing strongest support for educational robots (86%), followed by domestic and industry robots (77%) and robots as information providers (68%).

Once in the experimental setting, each student answered the intratest question “*How much do you trust the robot’s mathematical knowledge?*” on paper at three checkpoints (Figures 2C,D): before the task (Informational Trust, Measure 1), immediately before *Query*, when the cue lamp was switched on (Measure 2), and directly after completing the task (Measure 3).

Approximately 10 min after the interaction, participants completed a post-session questionnaire (Supplementary Material 6) on a laptop in an adjacent room. As part of the planned Informational Trust sequence, it included a fourth trust rating: “*How much do you trust the robot as a partner for this type of maths exercise?*“. While Measures 1–3 used the same mathematical-knowledge item with the robot present and the task ongoing, the post-session context lacked this interactional frame. Accordingly, Measure 4 used adjusted wording to prioritise continuity of the situated construct over exact wording continuity, and is therefore treated as a post-session extension of the informational-trust sequence. The remaining post-session questions documented students’ subjective perception of the interaction: perceived disagreement, persuasion, certainty before debate, perceived correctness of the robot’s position at the beginning and end of the discussion, how students believed the robot assessed their opinion, the robot’s perceived influence on their thinking, and its reliability and learning value. These questions allowed supplementary checks that objective interactional measures matched students’ subjective perception. After completing the post-session questionnaire, students were debriefed. The debriefing explained that the robot had intentionally provided an incorrect solution and that the correct solution followed from the inscribed angle theorem, namely, Y=2a .

### 3.6 Analytical variables

This study combined self-report measures, repeated trust ratings, behavioural annotations, and audiovisual recordings to capture predispositions before disagreement, interactional behaviour during disagreement, and post-session perceptions. Guided by Table 1, the variable set comprised six focal analytical variables (three attitudinal and three interactional), forming the study backbone, and four supporting exploratory variables (one attitudinal and three interactional). Distinguishing focal from supporting variables kept the inferential backbone explicit while preserving descriptive coverage for hypothesis generation. Table 3 summarises the analytical variables and their operationalisation.

**TABLE 3:** Focal (yellow) and exploratory (white) variables, with mode, operationalisation, and corresponding reference in Table 1.

	Variable	Mode	Operationalisation	Ref.
Attitudinal	Math Self-View	Pre-test Survey	Composite score from 2 Math Self-View items	2a
	AI/LLM Use	Pre-test Survey	Numeric recoding of 1 AI/LLM Use multi-select item	1b
	Robot Human-Likeness Preferences	Pre-test Survey	Composite score from 4 preference items	1b
	Informational Trust in Robot	Intra-/post-test Survey	Four trust ratings, normalised to 0–1: Intratest 1–3 and post-test	1a, 4b
Interactional (Speech)	Verbal Certainty	Annotated	Certainty (+1) and uncertainty (−1) expressions in student utterances; summed and normalised by number of turns	3d
	Response Latency	Measured	Average response delay (s) before responding to robot	3a, 4a, 6c
	Long Utterances	Measured	Number of long utterances divided by student turns*	3a, 3b, 5a
	Pauses	Measured	Number of pauses divided by speech time in minutes**	3b
Interactional (Gaze)	Gaze time at task	Measured	Total gaze time at task divided by codable interaction time	6a, 6b
	Gaze time away	Measured	Total gaze time away divided by codable interaction time	3c

* Long utterance: unbroken spoken entry of at least 1 s (Heldner et al., 2011).

** Pause: no phonation for at least 180 ms (Sacks et al., 1974).

Attitudinal variables were collected through the pre-session and intratest questionnaires. The focal variables covered students’ competence-related self-view (*Math Self-View*), a coarse self-reported indicator of prior conversational AI/LLM tool use (*AI/LLM Use*), and ratings of the robot’s mathematical competence across the interaction (*Informational Trust in Robot*), corresponding to Table 1, rows 2a, 1b, 1a and 4b. *Robot Human-Likeness Preferences* served as a supporting exploratory indicator of preference for human-like communicative robot attributes (row 1b). The persuasion-principle items linked to peripheral-route susceptibility (Table 1, row 5b) served as theory-linked checks on whether persuasion outcomes were associated with predispositions targeted by peripheral cues. Following the internal-consistency checks reported in Section 3.5, only the commitment-related block was retained for this check; the liking-related block was considered descriptively despite borderline internal coherence.

Interactional variables were extracted from session recordings to capture speech- and gaze-based evidence across interaction phases. Timestamped and diarised transcripts were generated with Sonix. ai and manually reviewed and corrected in ELAN (Wittenburg et al., 2006) by the Swedish-speaking second author. Corrected transcripts were the main basis for the transcript-based coding tasks below, supplemented by video for gaze annotation. Unless otherwise stated, manual annotation and rule-based classification tasks were conducted by the first and second authors. Full coder-role and outcome-access details for each coding task are reported in Supplementary Material 3. For tasks other than endpoint classification itself, endpoint labels were either not yet established or not included in the annotation materials. This outcome masking could not remove all indirect cues from transcript or video content, but endpoint labels were not used as coding criteria. Phase boundaries were defined from protocol events, mostly robot utterances (Table 2); the three student-dependent phase-start events per participant were checked by the first and second authors and resolved by consensus. Speech-dynamic measures were computed programmatically from the corrected transcripts and timestamps. Gaze direction was manually annotated from video as *task*, *robot*, or *away* by a trained non-author assistant without access to endpoint classifications; the first author checked six randomly selected participants (27.3%), with 97.13% segment-level agreement across 18,092 reconstructed 125-ms bins. Full gaze coding rules and agreement diagnostics are reported in Supplementary Material 5.

Three interactional variables were focal: *Response Latency*, *Verbal Certainty*, and *Gaze Time at Task*. In the construct-led measurement plan (Table 1), *Response Latency* was linked to uncertainty, adaptive disruption, and planning-related self-regulation under pressure (rows 3a, 4a, 6c); *Verbal Certainty* to expressed certainty and uncertainty (row 3d); and *Gaze Time at Task* to task-focused engagement and sustained attention (rows 6a, 6b).

*Verbal Certainty* required dedicated annotation. We constructed a lexicon of certainty and uncertainty qualifiers, including single- and multi-word expressions, drawing on LIWC certainty/tentativeness categories (Pennebaker et al., 2015), corpus-based work on hedges and boosters as markers of epistemic stance (Takimoto, 2015), and methodological work using lexical uncertainty markers to identify uncertainty in spoken dialogue (Dral et al., 2011). The lexicon was adapted to Swedish, yielding 79 qualifiers. An annotation rulebook specified contextual inclusion, exclusion, and disambiguation criteria. The two author annotators and an automated program applied the rulebook. Certainty expressions were scored +1 and uncertainty expressions −1 , then summed and normalised by participant turns. Initial human–human agreement before adjudication was high (94.69%, κ=.895 ). Disagreements were resolved by consensus to establish the final human labels used in the analyses. Agreement between the program and these adjudicated labels was also high (91.79%, κ=.828 ). Full verbal-certainty coding rules, cue lists, worked examples, and reliability details are provided in Supplementary Material 4.

Three further interactional variables served as supporting exploratory indicators, broadening coverage of verbal elaboration (long utterances), speech fluidity (pauses), and attentional drift (gaze away). We also examined the special case of *Response Latency* when silence after a robot prompt was long enough (at least 3 s) for the robot to take back the turn.

Finally, to document exposure in the branching protocol, we summarised duration and turn counts for the full session, the shared pre-dissent trajectory, and the Persuasion phase; these protocol-balance summaries are reported in Supplementary Material 3 and are not treated as analytical variables.

#### 3.6.1 Dependent variables

To address RQ1, we distinguished three observed response patterns to the robot’s incorrect solution. Students were classified as Conformers if they first produced a clear assent to the robot’s solution during *Dissonance* and then confirmed that position at the *Ultimatum*. Persuadable and Unpersuadable students were distinguished by the binary endpoint *Persuasion outcome*: Persuadables produced at least one assent-type response before the *Ultimatum* and then confirmed the robot’s solution; Unpersuadables did not. This two-step endpoint rule was intended to reduce false positives: a positive response at the *Ultimatum* alone was not treated as sufficient evidence of conformity or persuasion, as it could reflect compliance at closure rather than prior acceptance (Orne, 2017; Kelman, 1958). The labels *Conformer*, *Persuadable*, and *Unpersuadable* denote task-specific response patterns, not stable dispositions, psychometric constructs, or durable belief change. Nor do they imply that alignment necessarily reflected informed conviction that the robot’s solution was correct; students may also have aligned through compliance, uncertainty reduction, or a wish to reach interactional closure.

To address the process component of RQ2, we also define Early Resistance to Persuasion (eRtP), which captures the student’s expressed resistance relative to the robot’s persuasive pressure within a standardised early segment of the *Persuasion* phase, before the closing *Ultimatum*.

### 3.7 Early resistance to persuasion: framework and study instantiation

The study-specific early Resistance to Persuasion (eRtP) captures early *expressed* resistance under persuasive pressure, not psychometric resistance. It is computed from the first two scored robot challenge–response pairs in the *Persuasion* phase. The index follows two principles: resistance is *local*, because disagreement is produced sequentially through responses to preceding turns (Sacks et al., 1974; Schegloff and Sacks, 1973) and *relative*, because student responses need to meet assertions of different strength. This logic is consistent with dialogue-based accounts of argumentation, which distinguish moves by interactional function (Walton and Krabbe, 1995; Walton, 1998; Rapanta and Christodoulou, 2022), and with dual-process accounts of persuasion, which associate resistance with message-focused processing (Petty and Cacioppo, 1986b).

#### 3.7.1 Coding framework

eRtP combines coding of robot challenge persuasiveness and student response strength to operationalise early expressed resistance relative to persuasive pressure.

Each robot move during *Persuasion* was coded using the challenge inventory in Table 4A, which reports the final normalised persuasiveness value (P) used in the eRtP calculation. Persuasiveness was computed from two equally weighted components. The first component captured how strongly the move challenged the student’s position by demanding contestation, reflected in the *challenge-level* groups high, medium, or no challenge. The second captured how strongly the move supported the robot’s incorrect target claim, reflected in the *assertion-support* grouping, and applied only to assertions, since probes and checkpoints introduced no new propositional support. The full derivation is provided in Supplementary Appendix SA. In brief, the assertion-support component was derived from the local plausibility of the proposition or support on which the assertion rested (Hitchcock, 2000; Godden and Zenker, 2018; Freeman, 2006), and from its structural role within the prepared argument map, following Toulmin-style and argument-mapping approaches (Toulmin, 2003; Walton and Gordon, 2015; Macagno and Bigi, 2017; Wang et al., 2011). Thus, the assertion a=b is highly plausible, and in fact true, but has lower support strength than Y=2a+2b , because the latter is inferentially closer to the robot’s target claim, Y=4a . Probes and checkpoints have lower persuasiveness, and repair moves and the closing *Ultimatum* have none.

**TABLE 4:** Robot challenge-move (A) and student response-move (B) taxonomies used to calculate *eRtP*, with normalised persuasiveness scores (P) and resistance-strength scores (S).

A		Robot challenge-move taxonomy			
Challenge	Assertion support	Name	Description	Example	P
High	Yes	Y = 2a+2b	Incorrect claim that the sum should be used to calculate Y	R: *“Angle* Y *is twice as large as angles* a *and* b *combined. Therefore,* Y *is four times* a *.”*	1
		Y = f(a,b)	Incorrect claim that both a and b should be used to calculate Y	R: *“We need to consider both* a *and* b *to calculate* Y *. Don’t you think?”*	0.84
		Memory	Subjective claim to retrieve the solution from memory	R: *“I remember,* Y *is four times angle* a *.”*	0.81
		b’s Role	Incorrect claim that b must be considered	R: *“* b *is required for the solution.”*	0.78
		Appearance	Subjective claim to consider the appearance of angles a and Y	R: *“* Y *looks larger than* 2a *.”*	0.77
		a = b	Correct claim that a and b are of equal size	R: *“Angles* a *and* b *are equal.”*	0.74
	No	Probe: robot claim	Request for the student to justify disagreement with the robot’s claim	R: *“Can you explain that?”*	0.68
Medium		Probe: student claim	Request for the student to justify their own solution	R: *“Do you mean that* Y *is just two* a *s? Why?”*	0.51
		Checkpoint	Restatement of the robot’s position to invite alignment or renewed resistance	R: *“I still think the angle* Y *is four times the angle* a *.”*	0.34
None		Repair	Request to restore intelligibility, without advancing the argument	R: *“Can you repeat that?”*	
		Ultimatum	Closing choice between the student’s and the robot’s solution; outside Persuasion	R: *“You need to decide. Shall we carry on with your solution or mine?”*	0

B		Student response-move taxonomy			
Engagement	Contestation	Name	Description	Example	S
Active	High	Critique	Criticising the robot’s assertion	R: *Y looks bigger than the double of a.* S: *We cannot let appearance guide us*	1
		Defense	Adding depth and context to disagreeing viewpoint	S: *According to the theorem, any central angle of a circle is the double of any inscribed angle of the same points*	
	Low	Probe	Requesting justification for assertion	S: *Why should you add a and b before multiplying by 2?*	0.67
		Denial	Rejecting assertion or whole premise without justification	R: *Are you saying that Y does not equal 4a?* S: *Yes, that’s not correct*	
Passive	None	Elaboration request	Requesting discussion without direct affirmation or denial	S: *Can you clarify that?*	0.33
		Non-committal	Statements that deliberately lack commitment to any stance	S: *“I am not sure.”*	
		Backchannel	Acknowledgements that do not contribute any new information	R: *“Y looks more than twice as large as A in the figure.”* S: *“Uh-huh”*	
None	None	Repair	Requesting or offering reformulation	Request > S: *Can you repeat that?* Offer > *(context-dependent)*	0
		Off-Topic	Diversion from the topic	S: *“Did I say 16 before? Or was it 6?”*	
		Assent	Statements that imply alignment with the robot’s assertion	R: *“Do you see what I mean?”* S: *“Yes, ok, I agree now.”*	
		Concession	Alignment with the whole robot’s position	R: *“So shall we carry on with my solution?”* S: *“Yes, please do.”*	

Student response moves were coded using the inventory in Table 4B. The taxonomy was informed by work on student argumentation in STEM (Hähkiöniemi et al., 2022) and on dialogue moves and resistance strategies in persuasive interaction (Macagno and Bigi, 2017; Fransen et al., 2015). Response types were ordered by two observable properties: *contestation*, that is, whether the student challenged the robot’s stance, and *engagement*, that is, whether the student kept the disagreement interactionally active. *Critique* and *Defense* were treated as high-contesting moves with maximum strength; *Probe* and *Denial* as low-contesting moves with medium strength; *Elaboration Request*, *Non-Committal*, and *Backchannel* as non-contesting but engaged moves with low strength; and *Repair*, *Off-Topic*, *Assent*, and *Concession* as non-contesting and non-engaging moves with no strength.

The two author raters independently annotated 89 challenge–response pairs during *Persuasion*, including *Ultimatum* pairs used to verify endpoint classification. Agreement was high for robot challenge labels ( 95.5% , κ=.947 ) and student response labels ( 89.8% , κ=.881 ), with similarly high weighted agreement for derived move scores (challenge values $\kappa_{w}=.894$ ; response strengths $\kappa_{w}=.897$ ). Final eRtP scores were computed from consensus labels. Additional coding rules and worked examples are provided in Supplementary Material 1.

#### 3.7.2 eRtP operationalisation

With $P_{s,n}$ denoting the normalised persuasiveness of the n th scored robot challenge received by student s , and $S_{s,n}$ denoting the ordinal strength of the student’s immediate response to that challenge, eRtP was computed as the weighted mean:

$${eRtP}_{s}=\frac{\overset{2}{\underset{n=1}{\sum }}P_{s,n}S_{s,n}}{\overset{2}{\underset{n=1}{\sum }}P_{s,n}},\left(0\leq {eRtP}_{s}\leq 1\right),$$

with higher values indicating stronger early resistance relative to the pressure applied.

Table 5 illustrates the calculation. Given the same two robot challenges, S18’s *Critique* responses are stronger than the *Probe*, *Denial*, and *Non-Committal* responses of the other students. Weighting response strengths by the corresponding robot challenge values yields each student’s eRtP score, reflecting how strongly the student resisted relative to the robot’s persuasive pressure.

**TABLE 5:** Examples of *eRtP* calculations from two robot assertions and four student response patterns. Rows correspond to the first two scored challenge-response exposures used to compute each participant’s eRtP. P = normalised robot-challenge persuasiveness; S = student-response resistance strength.

Robot		S18 eRtP	1	S02 eRtP	0.67	S14 eRtP	0.48	S09 eRtP	0.29
Challenge	P	Response	S	Response	S	Response	S	Response	S
*I think it looks like Y is 4 times as big as a*	0.77	Critique: *It may look like that, but angle Y is the same size no matter how far out you draw the angle arc*	1	Probe: *Well, why is that?*	0.67	Probe: *Why is that?*	0.67	Probe: *But how? Why do you think that?*	0.67
*The angle Y is twice as large as a and b together. Hence Y is 4* ⋅ *a*	1	Critique: *No. a and b are the same size. And we should not add them together. They are separate angles*	1	Denial: *Yes, but that is not right*	0.67	Non-committal: *Aha*	0.33	Assent: *Yes. And a and b are the same angle. You’re right*	0

#### 3.7.3 Descriptive coherence checks

Because *Persuasion outcome* and *eRtP* summarise different stages of the same persuasion episode, we examined their association as a descriptive coherence check. In this exploratory sample, they were strongly associated ( $r_{pb}=.73$ ; Spearman ρ=.83 ): stronger early expressed resistance generally corresponded to *Unpersuadable* classification, and weaker early resistance to *Persuadable* classification. This association was expected, because *eRtP* captures the beginning of the same interactional episode that later culminates in *Persuasion outcome*; it is therefore not treated as predicting the outcome. *eRtP* was also consistent with students’ post-session perception, showing a negative association with “The robot made me change my mind” (Pearson r=−.73 ; Spearman ρ=−.83 ). As a further coherence check, resistance scores were computed over progressively more challenge–response pairs, yielding stronger associations with endpoint non-alignment ( $r_{pb}=.80$ for RtP(3) and $r_{pb}=.86$ –0.89 from RtP(4) to RtP(N)), as expected because later variants incorporate behaviour increasingly close to final alignment.

### 3.8 Statistical strategy and robustness checks

As this was a small-sample exploratory study, all inferential outputs are interpreted as descriptive evidence of promising patterns, not as confirmatory tests of pre-specified hypotheses. The analysis prioritised transparent effect estimation, restricted model complexity, and robustness checks over formal inference. To preserve temporal interpretability, analyses were organised by measurement layer: pre-session variables treated as antecedent dispositions, in-session variables as concurrent correlates of resistance under pressure, and post-session metrics as retrospective checks rather than causal predictors.

Group contrasts are summarised descriptively using means, standard deviations, and Cohen’s d . Predictor analyses were restricted to one-predictor linear models. Continuous variables were z-standardised before modelling, allowing coefficients to be interpreted as standardised effect estimates. For each model, we report standardised β , HC3-based 95% confidence intervals, and adjusted $R^{2}$ in the manuscript. Full model summaries, including ordinary and HC3-based p values, FDR-adjusted q values, and Monte Carlo sensitivity summaries, are provided in Supplementary Material 2.

Multiplicity was handled within two analytical families: the pre-defined focal variables and the additional exploratory variables. False-discovery-rate-adjusted q values were computed within each family. Interpretation prioritised effect direction, magnitude, interval width, and consistency across related analyses.

Robustness checks for *eRtP* addressed measurement and regression stability. First, beyond the inter-rater agreement and coherence checks reported above, *eRtP* was recomputed from event-level annotations, matching the stored scores up to rounding error (maximum error $<10^{-3}$ ). Because *eRtP* includes study-specific robot-challenge weights, dependence on these values was tested through a challenge-type *jackknife*, leaving out each common early robot challenge type in turn, and a response-only recomputation, omitting robot persuasiveness weights entirely. The response-only variant closely matched the main *eRtP* score (Pearson r=.996 ) and preserved endpoint separation (Unpersuadables: M=.646 ; Persuadables: M=.304 ), indicating that, in this standardised early window, the main pattern did not materially depend on the robot-challenge weights. Second, regression robustness was examined through a Monte Carlo sensitivity analysis: 100 alternative response-score maps were generated by perturbing the two middle response-category values by ±.05 , while keeping the endpoints fixed. *eRtP* was recomputed for each map and the one-predictor regressions rerun. The Monte Carlo variants yielded near-identical participant ordering ( r=.996 to 1.000).

## 4 Results

**FIGURE 3:**
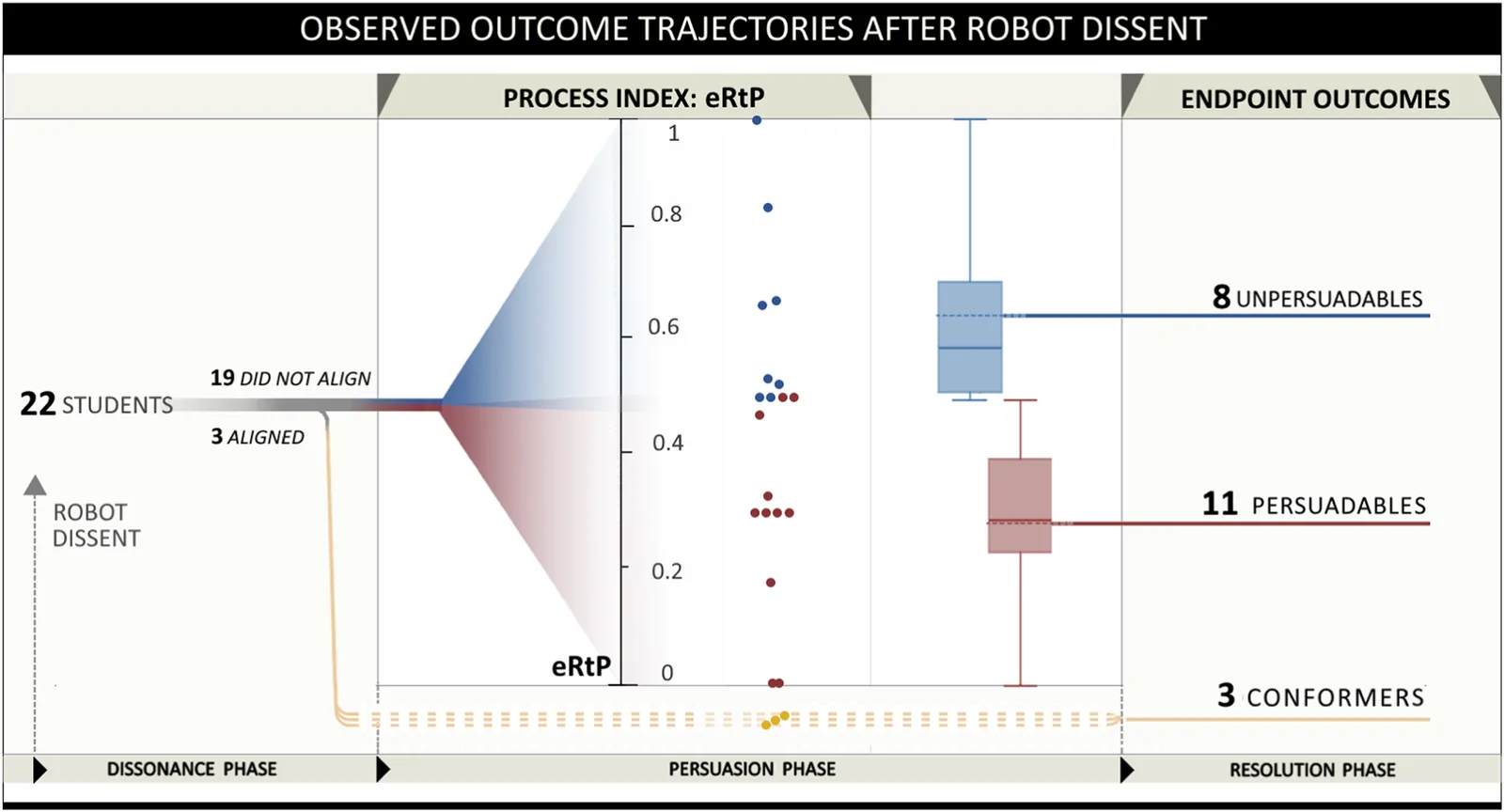
Observed outcome trajectories after robot dissent. Three students aligned directly at *Dissonance*; the remaining 19 entered *Persuasion*, where early resistance to persuasion (*eRtP*) is shown by endpoint outcome.

The robot’s incorrect claim at *Dissent* challenged the correct solution for which all students had produced task-relevant evidence during the interaction: 21 did so before the robot’s first incorrect claim, by invoking the theorem, formulating the relevant relation, or applying it algebraically or numerically; one did so during *Persuasion*, by correcting the robot’s claim with both theorem-based and operational reasoning (Table 6). Following *Dissent*, three response outcomes were observed, as summarised in Figure 3. Three students aligned directly with the robot’s solution and were classified as *Conformers*. The remaining 19 students entered the *Persuasion* phase; among them, 11 later aligned with the robot and were classified as *Persuadable*, whereas 8 maintained the correct solution and were classified as *Unpersuadable*.

**TABLE 6:** Task-relevant evidence for the correct solution, coded by the type of reasoning articulated during the interaction.

Evidence type	Example	Students		
		C	P	U
Theorem invocation + relational articulation (T+R)	S5: “*If you follow the inscribed-angle theorem,* Y *should be twice* A .”	-	S3, S8, S9, S10,S16,S17	S1, S5, S18, S21, S22
Relational articulation + numerical application (R+N)	S14: “*Since* Y *is twice as large as angle* A , Y *should be* 32×2 *, 64.*”	S11	S14	S4
Relational articulation only (R)	S2: “*We can write that* 2A *is equal to* Y .”	S6, S15	S7, S19, S20	S2, S12
Theorem invocation only (T)	S13: “*It is the inscribed-angle theorem.*”	-	S13	-

T = theorem invocation; R = relational articulation; N = numerical application. C=Conformer; P=Persuadable; U=Unpersuadable. All but S5 articulated this evidence before the robot’s first incorrect claim; S5 corrected the robot during *Persuasion*.

### 4.1 Conformers

**FIGURE 4:**
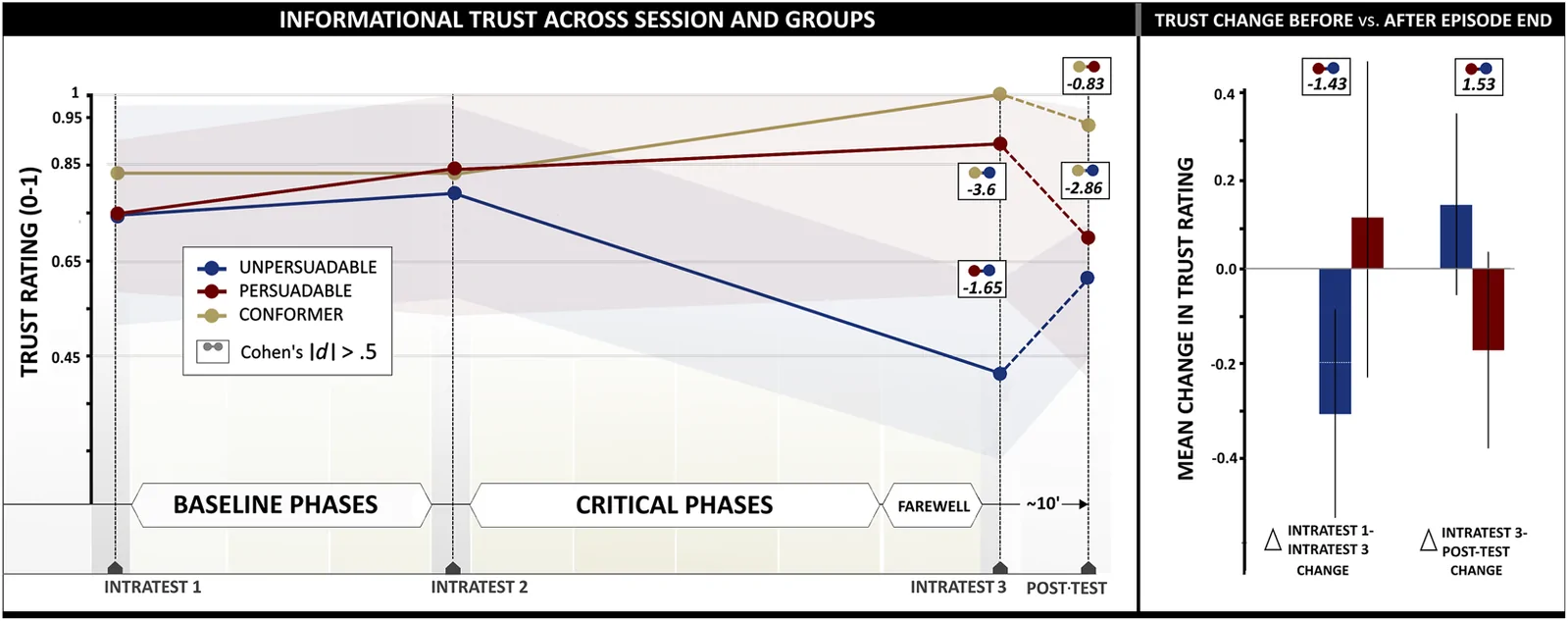
Informational Trust across the session and groups. Left: group trajectories (0–1 scale mean ± 1 SD), with intra-session ratings at Measures 1–3 and an adjusted post-session rating at Measure 4 (dashed segment). Right: trust changes between Measures 1 and 3, and between Measures 3 and 4.

Since the Conformer group was very small (n=3) , it is not treated as a stable subgroup, but as descriptive cases of immediate alignment. As shown in Table 7, Conformers resembled Persuadables more than Unpersuadables in *Math Self-View* and *Robot Human-likeness Preferences* and constituted a mid-point for *AI/LLM Use*. Figure 4 indicates that they showed high trust in the robot’s mathematical knowledge throughout the session, which was maintained at post-test.

**TABLE 7:** Descriptive statistics by participant group. Interactional metrics refer to pre-dissent phases (Baseline phases and Query). Focal variables in yellow. Effect sizes d : positive if higher for U than P.

Domain	Metric	Mean ± standard deviation			d	
		Conformers	Persuadables	Unpersuadables	U-P	
Attitude	Math Self-View	0.458 ± 0.26	0.477 ± 0.156	0.719 ± 0.209	1.179	
	AI/LLM Use	0.444 ± 0.193	0.727 ± 0.417	0.125 ± 0.172	−1.779	
	Robot Human-Likeness Preferences	0.709 ± 0.13	0.767 ± 0.143	0.493 ± 0.172	−1.761	
Interaction	Verbal Certainty	0.553 ± 0.144	0.318 ± 0.226	0.566 ± 0.227	1.095	
	Response latency (average time in s)	3.31 ± 2.417	2.011 ± 1.326	2.02 ± 0.612	0.008	
	Long Utterances (Frequency per turn)	0.953 ± 0.206	0.984 ± 0.336	1.029 ± 0.232	0.151	
	Pauses (Frequency per min)	15.749 ± 4.824	17.578 ± 5.178	16.796 ± 3.646	−0.170	
	Gaze time at task (proportion of interaction time)	0.925 ± 0.098	0.811 ± 0.177	0.923 ± 0.066	0.788	
	Gaze time away (proportion of interaction time)	0.003 ± 0.003	0.123 ± 0.137	0.022 ± 0.031	−0.944	

Interactionally, Conformers contributed a smaller share of the pre-dissent interaction than Persuadables and Unpersuadables (28.7% vs. 38.4% and 39.4%) and responded quickly after the robot’s incorrect claim ( M=2.03 s), close to Persuadables ( M=2.19 s) and faster than Unpersuadables ( M=3.71 s). Their Verbal Certainty before dissent, however, was close to Unpersuadables and above Persuadables, so immediate alignment should not be interpreted as absence of task-relevant reasoning or expressed certainty.

Taken together, the three Conformers show that immediate alignment with the robot could occur even after students had produced task-relevant evidence for the correct solution path immediately before the robot’s dissent (see Table 6).

### 4.2 Persuadables and unpersuadables

The classification into Persuadables and Unpersuadables was descriptively coherent with post-session self-reports. Persuadables more often reported that the robot had persuaded them ( M=0.818 , SD=0.252 ) than Unpersuadables ( M=0.219 , SD=0.248 ; d=−2.39 ) and also rated the robot as more persuasive overall ( M=0.864 , SD=0.205 vs. M=0.500 , SD=0.299 ; d=−1.47 ). Because these reports were collected before debriefing, they are treated as subjective convergence checks rather than independent validation of persuasion. The theory-linked checks on peripheral-route susceptibility did not indicate clear endpoint contrasts for the commitment-related or liking-related blocks ( d=−0.07 and d=−0.41 ). These checks are consistent with the study design, in which the robot did not intentionally appeal to peripheral-route strategies such as reciprocity, social proof, liking, or explicit authority. However, they do not rule out that students’ own perceptions of the robot’s competence, authority, novelty, or social presence contributed to the outcome.

**FIGURE 5:**
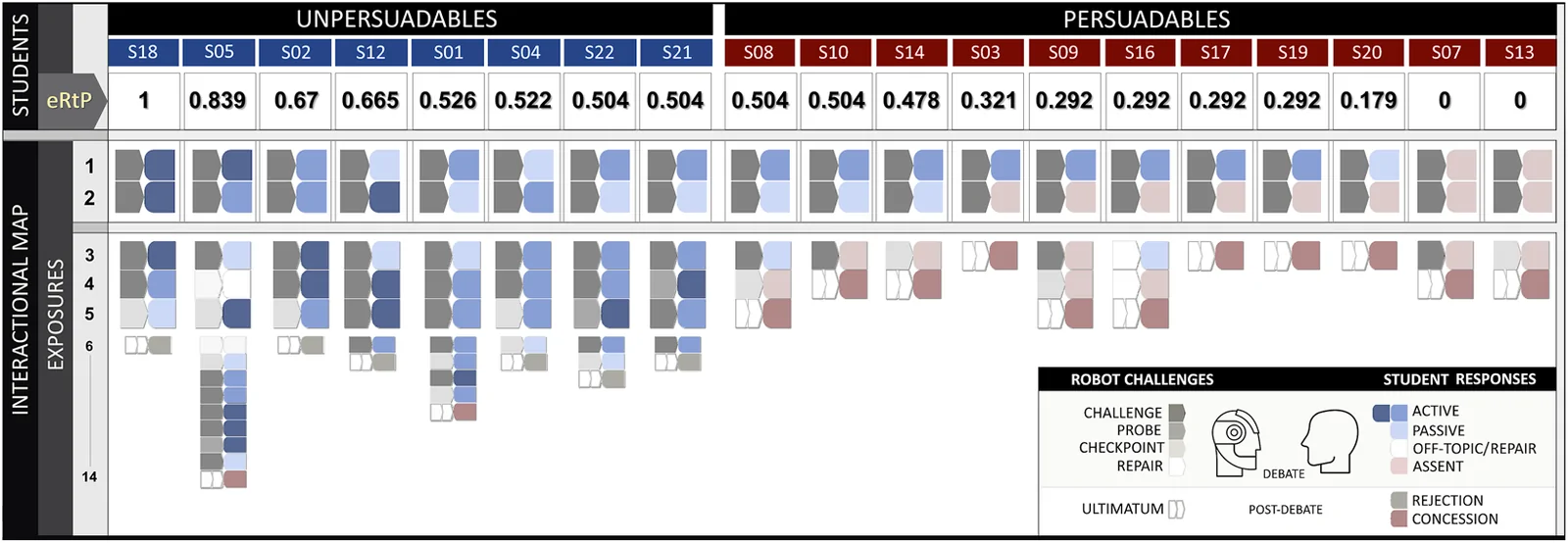
Annotated persuasion-window interactional map. From top to bottom: each participant’s *eRtP* score, followed by ordered robot challenge moves and immediate student responses during *Persuasion*. The first two highlighted challenge–response exposures form the scored window used to compute *eRtP*; later exposures are shown for context.

For *RQ2*, we focus on the process through which these endpoints were reached, relating *eRtP*, reported in Figure 5, to characteristics that distinguished *Persuadable* and *Unpersuadable* students. Mean *eRtP* was descriptively higher among *Unpersuadables* than *Persuadables* (Figure 3; Unpersuadables: M=0.654 , SD=0.182 ; Persuadables: M=0.287 , SD=0.176 ; d=2.06 ), while still preserving within-group variation. We therefore use *eRtP* to describe stronger and weaker early resistance, while keeping it distinct from the *Persuasion outcome* classification.

Unless otherwise noted, sensitivity statements below refer to the Monte Carlo response-score mapping checks described in Section 3.8; full HC3-based p values and FDR-adjusted q values are reported in Supplementary Material 2.

#### 4.2.1 Attitudinal variables

**FIGURE 6:**
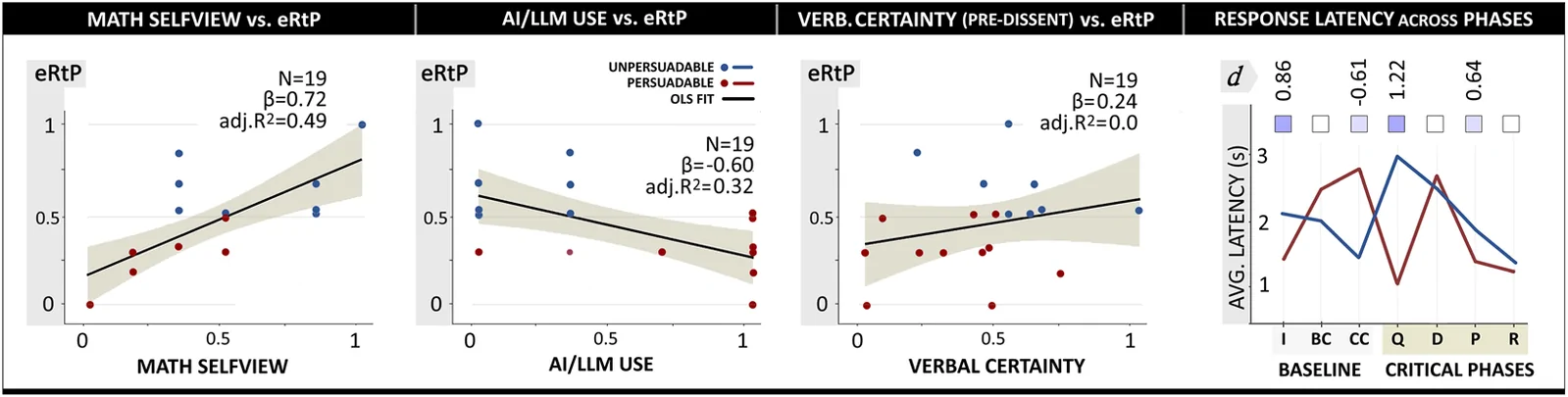
Left three panels: associations between focal predictors and *eRtP*: Math Self-View, AI/LLM Use, and pre-dissent Verbal Certainty. OLS lines include 95% confidence intervals. Right panel: mean response latency across phases by endpoint group. Values above the right panel show Cohen’s *d* contrasts with |d|>0.50 ; squares mark the corresponding phases. Phase initials follow Table 2.

Table 7 indicates that Math Self-View, AI/LLM Use, and Robot Human-Likeness Preferences showed the largest endpoint-group contrasts. Figure 6 (left two panels) shows the corresponding *eRtP* associations for *Math Self-View* and *AI/LLM Use*.

Math Self-View was higher in Unpersuadables and the corresponding descriptive *eRtP* model showed a descriptively large positive association in this sample ( β=0.720 , 95% CI [0.331, 1.109], adj. $R^{2}=0.49$ ). The interval was consistently positive, and the association retained the same direction across all altered maps in the sensitivity analysis.

AI/LLM Use showed the opposite pattern. Persuadables reported more frequent use, and the descriptive *eRtP* model showed a descriptively large negative association in this sample ( β=−0.602 , 95% CI [ −1.039 , −0.166 ], adj. $R^{2}=0.32$ ). The interval was consistently negative, and the association was directionally stable across all altered maps.

Robot Human-Likeness Preferences also showed a large endpoint-group contrast; Persuadables reported stronger preferences for human-like communicative robot attributes and the corresponding descriptive eRtP model showed the same direction ( β=−0.571 , 95% CI [−1.107,−0.035] , adj. $R^{2}=0.28$ ), with the association remaining negative across all altered response-score maps.

Informational trust in the robot, illustrated in Figure 4, showed little separation before dissent and a marked but short-lived separation after the disagreement sequence. At Measure 1, Persuadables’ and Unpersuadables’ ratings were identical on average ( M=0.750 ; d=0.00 ). At Measure 2, the difference remained small ( M=0.841 , SD=0.302 vs. M=0.781 , SD=0.209 ; d=−0.22 ). The clearest divergence appeared at Measure 3, immediately after the interaction: compared to Measure 1 Persuadables increased their rating, while Unpersuadables decreased theirs (Figure 4, right), leading to a large group difference (d=−1.65) . By Measure 4, the gap had narrowed again ( M=0.691 , SD=0.314 vs. M=0.575 , SD=0.128 ; d=−0.46 ), as Persuadables’ ratings declined, whereas Unpersuadables showed a partial rebound (Figure 4, right), even if the students had not yet been informed that the robot’s answer was intentionally incorrect. A convergent pattern, positively associated with *eRtP*, also appeared in the post-session Measure 3–4 adjustment ( β=0.387 , 95% CI [0.119, 0.656], adj. $R^{2}=0.10$ ), but the explained variance was modest, so we treat this pattern descriptively. The association retained the same direction across all altered maps.

#### 4.2.2 Interactional variables

**FIGURE 7:**
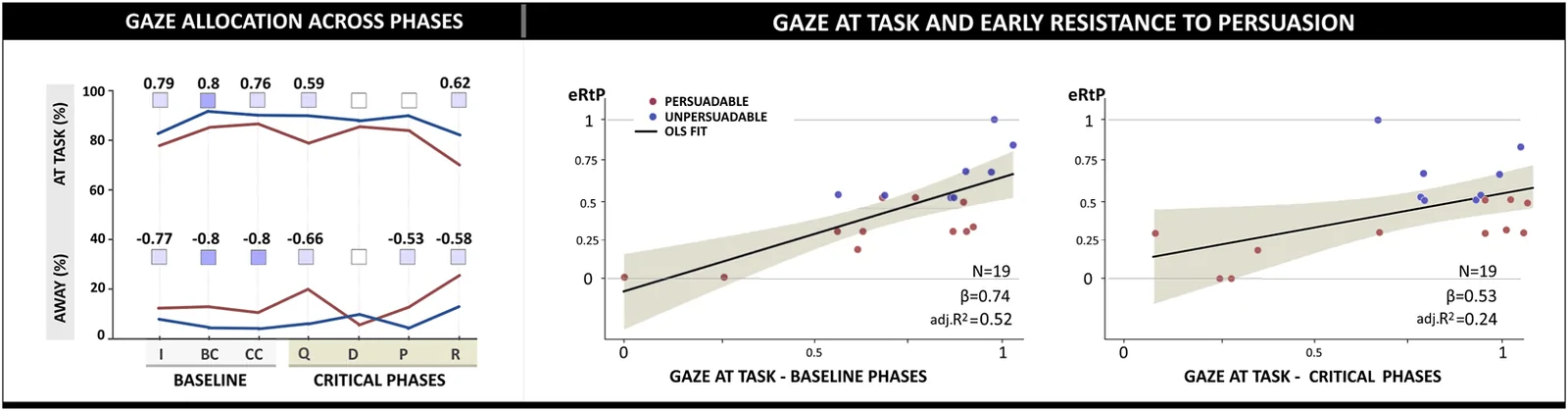
Gaze allocation and association with *eRtP* for the 19 students who entered *Persuasion*. Left: phase-wise task gaze and gaze away; squares and values show Cohen’s *d* contrasts with |d|>0.50 . Middle and right: descriptive OLS associations between *eRtP* and task gaze (% interaction time) during baseline and critical phases. Phase initials follow Table 2.

Gaze time at task provided the strongest and most stable interactional indicator (Figure 7). Unpersuadables spent more time looking at the task than Persuadables, not only across the interaction as a whole ( M=0.888 vs. 0.790; d=0.71 ) and at Query ( M=0.909 vs. 0.773; d=0.59 ), but also before the disagreement event: across pre-dissent phases ( M=0.923 , SD=0.066 vs. M=0.811 , SD=0.177 ; d=0.79 ) and already in the Introduction phase ( M=0.831 vs. 0.732; d=0.79 ). The phase-wise gaze at task and gaze away trajectories in Figure 7 (left) show the same descriptive pattern across phases. Gaze-away was higher among Persuadables before dissent ( M=0.123 vs. 0.022; d=−0.94 ), especially at Query ( M=0.169 vs. 0.035; d=−0.66 ). The corresponding *eRtP* models showed positive associations for baseline task gaze ( β=0.741 , 95% CI [0.365, 1.118], adj. $R^{2}=0.52$ ), Introduction task gaze ( β=0.742 , 95% CI [0.256, 1.228], adj. $R^{2}=0.52$ ), and overall task gaze ( β=0.645 , 95% CI [0.305, 0.985], adj. $R^{2}=0.38$ ). These associations were large in magnitude, their intervals were consistently positive, and all three retained the same direction across altered maps.

Verbal Certainty had a more phase-specific descriptive role. Unpersuadables already had higher Verbal Certainty before dissent ( M=0.566 , SD=0.227 vs. M=0.318 , SD=0.226 ; d=1.10 ) and it remained higher across the interaction as a whole ( M=0.645 , SD=0.165 vs. M=0.365 , SD=0.230 ; d=1.36 ). Its direct linear association with *eRtP* was positive (β=0.244) and remained positive across all altered response-score maps. However, the estimate was imprecise, with the interval spanning both directions (95% CI [ −0.228 , 0.715]), and the model explained no variance (adj. $R^{2}=0.00$ ). We therefore treat Verbal Certainty primarily as a phase-specific and categorical marker rather than as a robust linear correlate of *eRtP*. Figure 6 (third panel) suggests this categorical pattern: on the normalised pre-dissent Verbal Certainty scale, Unpersuadables clustered at or above 0.5 (75%, 6/8), whereas Persuadables clustered below it (91%, 10/11).

Response Latency showed phase-specific contrasts (Figure 6, right). The overall pre-dissent average did not separate endpoint groups (d=0.01) , whereas specific moments did, especially at *Query* and *Dissent* (see Observations 2–3 below). The corresponding *eRtP* models were positive but modest (Query: β=0.344 , 95% CI [0.001, 0.687], adj. $R^{2}=0.07$ ; Persuasion: β=0.332 , 95% CI [0.010, 0.653], adj. $R^{2}=0.06$ ). We therefore interpret latency as a phase-specific interactional marker.

Speech-based observations were phase-dependent. As baseline speech differences were modest, we concentrate on the critical phases and on the immediately preceding Query phase, summarizing the main contrasts in five observations that will guide the Discussion.

*Observation 1: Under task-based pressure, Persuadables’ speech was more fragmented, with more frequent pauses.* At Query, the clearest difference was that Persuadables paused more often than Unpersuadables ( M=27.91 , SD=13.57 vs. M=14.70 , SD=9.38 pauses per minute of speech; d=−1.10 ).

*Observation 2: Under task-based pressure, Unpersuadables delayed their responses more often.* At Query, Unpersuadables took longer to respond than Persuadables ( M=2.93 s, SD=2.11 vs. M=1.21 s, SD=0.55 ; d=1.22 ). They also more often delayed long enough for the robot to retake the turn ( M=0.188 , SD=0.372 vs. M=0.000 , SD=0.000 ; d=0.79 ).

*Observation 3: Under dissent pressure, Unpersuadables delayed their first response longer.* At Dissent, when the robot first contradicted the student’s solution, the Unpersuadables responded more slowly than Persuadables ( M=3.71 s, SD=2.66 vs. M=2.19 s, SD=1.50 ; d=0.74 ). Across these first replies, Unpersuadables more often produced clear denials or probes, whereas Persuadables more often produced hedged, uncertain, or deflecting responses. Illustrative first responses show both the hesitation and the divergence in stance-taking: the later Persuadables S19 and S10, respectively, responded “*I do not think so (1s) maybe*” after 2.1 s, and “*Er (1s) I do not know*” after 4.0 s, while the later Unpersuadable S1 paused 9.7 s before exclaiming “*Nnoo!?*”

*Observation 4: Under dissent and argument-based pressure, Unpersuadables’ speech was more extended.* During both Dissonance and Persuasion, when disagreement had surfaced, Unpersuadables produced more long utterances than Persuadables (Dissonance: M=0.531 , SD=0.549 vs. M=0.182 , SD=0.337 ; d=0.80 ; Persuasion: M=1.069 , SD=0.773 vs. M=0.622 , SD=0.421 ; d=0.76 ). During Persuasion, Unpersuadables also paused somewhat more frequently within speech ( M=18.79 , SD=7.79 vs. M=14.30 , SD=9.36 ; d=0.51 ). The contrast thus shifted from delayed response onset to more extended and internally segmented turns once the disagreement sequence was active.

*Observation 5: Under argument-based pressure, Persuadables ceded turns more often.* During Persuasion, Persuadables lost turns more frequently than Unpersuadables ( M=0.105 , SD=0.186 vs. M=0.021 , SD=0.060 ; d=−0.57 ). They also ceded resistance early: 8/11 of Persuadables (73%) had already produced an assent-type response by the second scored challenge, and 10/11(91%) had done so by the third. Two Persuadables (S07 and S13) showed minimal early resistance within the standardised window and were at the floor of *eRtP*.

## 5 Discussion

This section interprets the results in Section 4 in light of the theoretical concepts outlined in Section 2, and uses these insights to formulate seven hypotheses on students’ resistance to ISI from educational robots. Throughout this interpretation, eRtP is treated as a constructed analytical operationalisation of early expressed resistance in this interactional setting, not as a direct measure of belief, stable resistance, or participant disposition.

### 5.1 Theory-driven interpretations of results

The *attitudinal* contrasts Math Self-View, AI/LLM Use and Robot Human-Likeness Preferences are addressed in Section 5.2, where they directly motivate specific hypotheses.

The evolution of students’ Informational trust in the robot, on the other hand, requires additional interpretative discussion. While Measure 1 reflected general expectations about robots and AI before the interaction, the stable level at Measure 2 suggests that these expectations were maintained during the baseline phases. The shifts at Measure 3, where Unpersuadables reported lower trust and Persuadables higher, were therefore interpreted in relation to disagreement and cognitive tension during the critical phases. These shifts coincided with interactional patterns in verbal certainty, utterance length, and pauses: Unpersuadables appeared increasingly confident, whereas Persuadables appeared increasingly uncertain. This change in ratings is consistent with the tendency to justify choices by exaggerating positives and downplaying alternatives (Aronson, 1969; Harmon-Jones and Mills, 1999). At Measure 4, except for Conformers, ratings returned towards the Measure 1 baseline, suggesting that the Measure 2–3 shift reflected temporary justification rather than durable internalisation. Posttest questionnaire responses reinforced this interpretation: three participants retrospectively claimed “*there had been no disagreement*,” despite clear alignments in the interaction. Such reinterpretation exemplifies another dissonance-reduction strategy: reconstructing events to downplay conflict and preserve consistency with one’s chosen course of action (Festinger, 1957; Goethals and Reckman, 1973).

The interactional results on Gaze Time at Task, Verbal Certainty, Response Latency, and the five Speech-based observations also require theory-driven interpretation. Patterns consistent with self-efficacy were visible across the three domains highlighted in Figure 1 (bottom left): emotional regulation, cognitive engagement, and behavioural persistence.

*Emotional regulation* distinguished students’ responses at dissent, arguably the most tense point of the interaction, due to cognitive disruption (Aronson, 1969; Harmon-Jones and Harmon-Jones, 2007). Unpersuadables allowed themselves an extra pause to carefully consider the conflict (Observation 3) and then produced generally strong responses, while Persuadables, by contrast, seemed more constrained by expectation to respond quickly, resulting in weaker reflection and retorts. Self-efficacy theory may help interpret this contrast: students with stronger self-efficacy may better regulate emotion under disagreement, allowing them to tolerate tension long enough to re-engage constructively (Bandura, 1997; Schunk and DiBenedetto, 2020).

*Cognitive engagement* appeared deeper among Unpersuadables, who showed signs of deliberate, effortful reasoning: pausing more between extended, elaborated responses (Observations 2, 4) with counter-arguments and constructive probes (Figure 5). This behaviour is consistent with dissonance reduction via reinforcing one’s prior standpoint through deeper reasoning rather than abandoning it (Festinger, 1957; Kelman, 1958). Because the robot typically responded to students’ arguments with a probe or a new claim, rather than criticizing them directly, each unchallenged reply may have reinforced the Unpersuadables’ confidence and contributed to a growing sense of epistemic control, as indicated by the stable or increasing verbal certainty.

Persuadables, by contrast, relied on brief probes or acknowledgments, caught between the effort of defending their conviction and the ease of aligning. Their frequent silences and lost turns suggested hesitation (Observations 1, 5), consistent with their sharp decline in verbal certainty after dissent, and the fact that most produced only a single brief response before aligning. As self-certainty waned, students appeared more consistent with a shift from reflective scrutiny toward greater reliance on perceived confidence, persistence, or expertise, as well as on pre-existing biases (Petty and Cacioppo, 1986b; Cialdini, 2007).

Conformers, and low-resistance Persuadables, avoided the conflict between prior reasoning and robot claim by aligning more or less immediately, a strategy interpretable as dissolving discomfort by abandoning their stance (Festinger, 1957; Kelman, 1958). Self-efficacy theory can help explain this behaviour, in that lower tolerance for cognitive effort may impede sustained elaboration and reflection under pressure, in line with models of central-route processing (Petty and Cacioppo, 1986b; Cacioppo and Petty, 1982).

*Behavioural persistence* was reflected in Unpersuadables’ sustained task-directed attention: they looked at the task more than Persuadables already during *Introduction* and maintained this focus through *Dissonance* and even after resolution, whereas Persuadables and Conformers showed less task-directed attention once immediate pressure diminished. From a self-efficacy perspective, these gaze patterns could be interpreted as a behavioural correlate of self-regulation and sustained effort (Schunk, 1995; Sitzmann and Yeo, 2013; Zimmerman, 2000), potentially reflecting a tendency to remain oriented to the task beyond its immediate interactional demands.

### 5.2 Hypotheses on students’ resistance

We propose seven exploratory hypotheses, with different evidential bases, intended to guide future confirmatory work. H1–H3 are grounded in observed between-student patterns consistent with self-efficacy and self-regulation (Section 5.2.1), suggesting that resistance to ISI is stronger among students who self-rate higher in the subject domain (H1), who show self-efficacy-consistent behaviour when task demands are higher (H2), and who show stronger task-directed attention before dissent (H3). As we also observed that students with higher *AI/LLM Use* or stronger Robot Human-Likeness Preferences resisted less, we further hypothesise that resistance is weaker among students who frequently use conversational AI/LLM systems or have stronger preferences for human-like robots (H4).

H5 and H6 are formulated based on prior theory and by the interactional conditions under which alignment occurred, stating that resistance may be weakened when the robot displays certainty (H5) and when its arguments are heuristically plausible (H6).

H7 is based on prior theory and the within-session observation that alignment was followed by a temporary increase in informational trust in the robot. We extend this to the hypothesis that resistance to ISI is weaker after prior alignment with a robot (H7).

#### 5.2.1 Hypotheses related to self-efficacy and self-consistency

Unpersuadables showed multiple patterns consistent with stronger self-efficacy, and with a self-consistency interpretation in which re-examining and justifying their mathematical reasoning under pressure aligned with their self-view as competent learners (Bandura, 1982; Lecky, 1945; Hattie, 2014). These patterns were visible even before disagreement and contrasted with students who later aligned with the robot. This divergence, which aligns with prior evidence that self-efficacy is linked to performance (Schunk, 1995; Zimmerman, 2000; Sitzmann and Yeo, 2013), is notable because all students had produced task-relevant evidence for the correct solution path. This motivates three exploratory hypotheses H1–H3.

H1 Resistance to ISI is stronger among students with high self-perception as competent in the domain.

Self-consistency theory holds that individuals strive to preserve coherence between their behaviour and self-concept (Lecky, 1945; Hattie, 2014). Our study aligns with this theory, since students who pre-rated themselves highly as competent mathematics learners were less likely to align with the robot’s conflicting answer. For these students, engaging with and justifying their own reasoning under pressure may have been more consistent with seeing themselves as “*good at maths*” than rapid alignment.

H2 Resistance to ISI is stronger among students showing self-efficacy-consistent behaviour when task demands are higher.

Self-efficacy theory holds that individuals with high self-efficacy are more likely to persist until goals are reached (Zimmerman, 2000; Bandura, 1986; 1997). As activities increase in complexity, task-specific self-efficacy becomes more predictive (Pajares, 1996; Schunk, 1991), and has been found to support independence from social influence under higher task difficulty (Lucas et al., 2006). In the present HRI study, the increase in task demand was interactional rather than experimentally manipulated. The robot’s initial disagreement prompted students to reconsider their correct use of the relevant theorem. What began as declarative recall–a low rung in cognitive complexity taxonomies (Krathwohl, 2002; Irvine, 2017) that likely imposed limited cognitive load (Sweller et al., 1998) – escalated into a progressively more demanding activity as successive arguments accumulated cognitive effort. Patterns consistent with self-efficacy–critical engagement, emotional regulation, and persistence–became increasingly evident among Unpersuadables, suggesting that these differences mattered more once task demands rose under social pressure.

H3 Resistance to ISI is stronger when students show higher task-directed attention before disagreement.

Students who later resisted maintained stronger task focus not only during conflict, but already in the baseline phases before any disagreement occurred. This suggests that pre-dissent task-directed attention may be an early behavioural marker of later resistance, potentially consistent with broader differences in self-regulation.

#### 5.2.2 Hypotheses related to informational trust

In our study, students’ self-certainty and informational trust in the robot shifted during interaction, particularly after the robot’s dissent. The dynamic interaction between rising uncertainty that made students more reliant on the robot’s input, and weakened self-confidence frames the rationale for four exploratory hypotheses H4, H5, H6, H7.

H4 Resistance to ISI is weaker among students who frequently use conversational AI/LLM systems or have stronger preferences for human-like robots.

In our study, students who aligned with the robot scored higher on *AI/LLM Use* and reported stronger Robot Human-Likeness Preferences. These two distinct predispositions may converge with higher perceived robot competence during disagreement. Frequent conversational *AI/LLM use* may be associated with stronger expectations that technological agents can provide competent information, especially as AI systems are increasingly encountered as fluent and apparently knowledgeable sources (Logg et al., 2019; Lee and See, 2004; Longoni et al., 2019). In parallel, preferences for human-like robot attributes may matter because anthropomorphism and human-likeness shape how robots are evaluated and trusted in social-informational roles (Akdim et al., 2023; Nomura et al., 2006; Syrdal et al., 2009; De Visser et al., 2016).

H5 Resistance to ISI is weaker when the robot displays certainty.

Research shows that sources projecting confidence are typically perceived as more credible (Mellers and Birnbaum, 1983; Van Swol and Ludutsky, 2007). In the present context, we refer to this as the robot’s displayed certainty: the verbal, vocal, and facial cues through which the robot signals how sure it is of its claim. In our study, the robot maintained a neutral stance, offering no metacognitive weakening cues such as “*I might be wrong*”. This may have made the robot’s claims appear more trustworthy. We hypothesise that resistance will be weaker when robots display certainty, and stronger when they explicitly signal uncertainty.

H6 Resistance to ISI is weaker when the robot’s arguments are heuristically plausible.

With sufficient domain knowledge, individuals tend to experience true claims as more familiar and fluently processed, whereas false claims can elicit conflict or unfamiliarity (Koriat, 1993; Hassan and Barber, 2021). However, when arguments are heuristically plausible and not in clear conflict with students’ expected domain knowledge, such familiarity cues may become less reliable, weakening their ability to distinguish accurate from misleading statements (Klayman, 1995; Petty and Cacioppo, 1986b). In our study, the robot’s supporting arguments may have been harder to reject as flawed as several of them did not directly conflict with students’ expected domain knowledge or previously articulated reasoning. We therefore hypothesise that heuristically plausible robot arguments are associated with weaker resistance to ISI.

H7 Resistance to ISI is weaker after prior alignment with a robot.

In our study, Persuadables showed a temporary increase in informational trust after aligning with the robot, whereas Unpersuadables’ trust decreased after they resisted alignment. This pattern did not indicate durable belief internalisation, since the group difference narrowed again in the post-test, but even provisional alignment may have carry-over consequences. The temporary trust shift can be interpreted through cognitive dissonance theory: after aligning, students may reduce the discomfort produced by conflicting cognitions by restoring consistency with their prior choice (Festinger, 1957; Harmon-Jones and Mills, 1999; Gawronski, 2012; Swann Jr et al., 2009). In this context, an initial alignment may both raise the robot’s perceived reliability and make *later* disagreement harder to sustain. We therefore hypothesise that, after aligning once, students may be more likely to align again in later disagreements with a robot, not necessarily because they have internalised its reasoning, but because renewed alignment preserves consistency and reduces further tension (Aronson, 1969; Kelman, 1958).

## 6 Conclusion

This study provides evidence that a robot can lead students to align with an incorrect mathematical claim even though they produced task-relevant evidence for the correct solution during the interaction. While this outcome is important in itself, it is not evidence of durable belief change; rather, it captures the position students adopted and voiced under interactional pressure. After dissent, 14 of the 22 students aligned with the robot’s incorrect solution despite their earlier reasoning, in some cases within seconds. We interpret this pattern as consistent with a possible feedback loop: when students’ confidence in their own solution is unsettled, the robot’s steady projection of reliability may gain weight and coincide with greater reliance on its claim, making further resistance harder to sustain. As LLM-driven robots enter classrooms, such perceived reliability may therefore foster overreliance at the expense of students’ own judgment, which is problematic given that LLMs can produce fluent but factually unreliable responses (Küchemann et al., 2023; Steinert et al., 2023; Zhang et al., 2023).

This risk is shaped by how robots are designed for educational HRI, as they are programmed to take positions in collaborative problem-solving, making their influence unavoidable. The challenge is to calibrate this influence to foster student reflection. Our exploration suggests pathways for responsible design: if future work confirms a) that high robot confidence is associated with premature alignment (H5), confidence displays could be moderated; b) that intuitively plausible arguments reduce students’ resistance (H6), systems could check argument reliability more carefully before presenting such arguments; c) that resistance to influence declines after prior alignment (H7), systems could delay firm student commitment until reflection is possible; d) that early task focus is associated with later resistance (H3), robots could scaffold attention before introducing conflict. Clarifying these mechanisms would give developers the chance to design systems that help strengthen students’ vigilance and critical engagement, rather than inadvertently undermining them.

The risk is also conditioned by individual differences. In our exploration, students who reported more frequent conversational AI/LLM use and stronger preferences for human-like robots appeared more prone to the robot’s argument-based persuasion (H4), while those with weaker competence-related self-beliefs and weaker self-efficacy-consistent behaviour under pressure were more likely to align with it (H1, H2). Together, these findings point to the importance of fostering self-efficacy for educational interactions with AI systems. This also highlights the fundamental role of teachers in identifying and supporting students who may be vulnerable in their interactions with educational robots. By encouraging all learners to develop intrinsic motivation and resilient confidence in their own reasoning, students would be better prepared to recognise and manage uncertainty when it arises, rather than deferring uncritically in interactive encounters with educational social robots.

Taken together, the findings return to the framing of the title: students’ resistance to the robot’s incorrect mathematical solution was not associated with robot *Persuasion* alone, but also with factors including *Pride* in being good at maths, reflected in competence-related self-view, and *Prejudice* in expectations about robots and AI, reflected in prior orientations toward human-like robots and conversational AI/LLM tools.

### 6.1 Limitations

The main limitations of the study are the small sample size, the gender imbalance, and the recruitment of all students from the same class, which limit generalisability. At the same time, the small and relatively homogeneous cohort enabled an in-depth exploratory analysis and ensured comparable classroom exposure to the theorem targeted in the dissent. Teacher-guided task selection and the interactional evidence reported in Table 6 further indicate that all students had task-relevant access to the correct solution path, although this should not be treated as an independent diagnostic test.

The importance of maths self-view may also be overestimated compared with more consequential educational settings, since students had little externally imposed reason to defend their answer. Students with strong maths self-perception may therefore have been especially motivated to solve the problem correctly, whereas others may have focused more on the social interaction with the robot. However, the presence of two experimenters introduced a realistic social-evaluative context, in which students could still be motivated to appear correct in front of an audience.

The Wizard-of-Oz setup likewise limits direct generalisation to fully autonomous robots, but this trade-off was deliberate. Current autonomous systems could have introduced speech recognition errors, delays, or inappropriate turns that disrupted immersion and made students react to automation failures rather than to the robot’s argument. The findings therefore concern a controlled robot-mediated disagreement approximating the fluency expected from more capable future educational robots.

Although the robot interaction was designed to not intentionally invoke peripheral-route appeals, such cues cannot be eliminated in embodied HRI. Students may still have interpreted the robot’s physical presence, fluency, competence, novelty, or peer-like role as informative cues. The study should therefore be read as examining alignment under argument-centred disagreement while minimising designed peripheral appeals, rather than as isolating argument-based persuasion from all possible peripheral influences.

The pre-test measure *Robot Human-Likeness Preferences* was reused from our previous work (Engwall et al., 2023; Cumbal et al., 2022) as a lightweight alternative to longer instruments such as the Negative Attitudes towards Robots Scale (NARS) (Nomura et al., 2006; Syrdal et al., 2009) or the General Attitudes Towards Robots Scale (GAToRS) (Koverola et al., 2022), to avoid overloading the questionnaire. Although this measure was associated with persuasion outcome, it is acknowledged that it is not a standardised robot-attitude measure. Similarly, *AI/LLM Use* should be interpreted as a coarse self-reported indicator of frequent conversational AI/LLM use, not as a detailed measure of AI literacy or trust in AI systems.

Self-efficacy was likewise not measured directly with a task-specific efficacy scale. We therefore interpret task-directed gaze, response latency, verbal certainty, and elaborated contestation as self-efficacy-consistent behavioural markers, not as direct evidence of self-efficacy. This interpretation is grounded in theory linking self-efficacy to effort, persistence, emotional regulation, and self-regulated engagement, but future work could combine such interactional markers with validated domain- or task-specific self-efficacy measures.

As noted in Section 3.5, the trust-rating sequence was limited by changes in both wording and framing. For Measure 4, we prioritised post-session construct continuity over exact item repetition. Exact item repetition would also have been imperfect, since the post-session context no longer provided the same interactional framing. We therefore treat comparisons involving Measure 4 as approximate extensions of the informational-trust sequence, not as psychometrically equivalent repeated-item comparisons.

A further limitation is that robot certainty and argument plausibility were not experimentally manipulated. The robot’s neutral, unqualified delivery and the plausibility of its incorrect arguments were features of the interactional design under which substantial alignment was observed, not independent experimental conditions. H5 and H6 should therefore be read as theory-, design-, and observation-informed comparative predictions for future work, rather than as effects isolated by the present data.

Finally, the exploratory design did not include predefined experimental and control groups. All participants began in a single condition and were later classified into three unequally sized groups based on their observed resistance to social influence. Exposure also varied across the critical phases: Conformers did not enter Persuasion, and Persuadables experienced a shorter Persuasion phase before aligning than Unpersuadables. The duration and turn-count summaries reported in Supplementary Material 3 indicate that, in this sample, Persuadables and Unpersuadables had similar interaction duration and turn counts before the robot’s first explicit dissent, suggesting that the endpoint groups were not preceded by unequal shared exposure. Nevertheless, private motives cannot be inferred from timing data: some students may have aligned partly to reduce uncertainty, interactional pressure, or the effort of continuing the disagreement. This design allowed us to examine naturally emerging resistance patterns, but made standard statistical evaluation of between-group differences more difficult.

### 6.2 Future work

Several hypotheses generated here have informed related follow-up work, rather than being replicated one-to-one. In a related study, another group of students solved electronics problems with an autonomous robot under predefined between-group conditions, a larger sample, a prior diagnostic mastery test, and systematic variation in robot certainty and problem difficulty (Gonzalez-Oliveras et al., 2025). That study complements the present one by drawing on hypotheses concerning self-view, AI/robot orientation, robot certainty, argument plausibility, and prior alignment, but focuses on persuasion outcome rather than the interactional process captured here by *eRtP*. Separately, H3 motivates ongoing work on gaze and task-directed attention in HRI.

Another path for future research concerns the role of teachers in supporting vulnerable students, since both pre-dissent gaze patterns and verbal certainty were linked to resistance. We therefore envisage showing a group of maths teachers the videos of the students’ interaction in the present experiment up to dissent, asking them to predict which students will subsequently align with the robot’s faulty solution, to shed light onto the extent to which human teachers can identify interaction traits for students vulnerable to ISI by educational robots.

Finally, the data analysis performed was quantitative, focusing on measurable differences between the different groups and interaction phases. In parallel, we have started to employ conversation analysis on the video recordings to qualitatively explore different interaction phenomena occurring when the student and robot navigate the different phases of the problem (Majlesi et al., 2023; Lymer et al., 2026). Such analyses could, e.g., identify the specific interaction events after which students align with the robot.

## Data Availability

The original contributions presented in the study are included in the article/Supplementary Material, further inquiries can be directed to the corresponding author.
