# Supplementary material for: Persuasion, pride and prejudice: factors associated with students’ resistance to a social robot’s incorrect mathematical solution
Source: Front Robot AI. 2026 Sep 10;13:1901093. doi: 10.3389/frobt.2026.1901093 (PMC13601342; doi:10.3389/frobt.2026.1901093)
Supplement: Supplementary file 1 [file Supplementaryfile4.pdf]

Supplementary Material 4
Verbal certainty annotation rules

Associated manuscript: *Persuasion, Pride and Prejudice: Factors associated with students' resistance to a social robot's incorrect mathematical solution*.
Purpose: This supplement documents the verbal certainty coding rules, cue lists, annotation procedure, reliability checks, and interpretation limits used to construct the Verbal Certainty variables. It is separate from Supplementary Material 1, which documents early Resistance to Persuasion (eRtP).

S1. Scope of this supplement

Verbal Certainty is an operational interactional measure of expressed epistemic stance in the student's speech. It captures explicit lexical or grammatical markers of certainty and uncertainty in student utterances. It does not measure private confidence directly, and it does not evaluate whether the student's mathematical answer was correct.

The coding procedure combined deterministic cue matching with human verification. Candidate expressions were identified from frozen cue lists and then reviewed by two human raters. Final labels were established after consensus on disagreements.

S2. Unit of analysis and temporal windows

Element	Operational definition
Coded unit	One student utterance, corresponding to one transcript row.
Speaker eligibility	Only student utterances were coded. Robot turns were ignored.
PRE-DISSENT window	Baseline phases plus Query.
POST-DISSENT window	Dissonance, Persuasion, and Resolution.
Label set	CERTAIN, UNCERTAIN, or NEUTRAL.
Per-utterance score	CERTAIN = +1, UNCERTAIN = -1, NEUTRAL = 0.
Aggregation	Scores were summed within a participant-window and normalised by the number of student turns in that window.

For participant s in window w, with utterance-level scores $v_i \in \{-1, 0, +1\}$ and $N_{s,w}$ student turns, the window-level score was computed as:

$$VC_{s,w} = \frac{\sum_{i=1}^{N_{s,w}} v_i}{N_{s,w}}.$$

Positive values indicate more expressed certainty, negative values indicate more expressed uncertainty, and values near zero indicate either few explicit markers or a balance between certainty and uncertainty cues.

S3. Frozen cue lists

The final cue lists were frozen before reliability scoring. Cues were matched case-insensitively. Certainty disqualifiers were applied before certainty qualifiers, so phrases such as *vet inte* were not misclassified as certainty because they contain *vet*.

Certainty disqualifiers	Certainty qualifiers	Uncertainty qualifiers
inte säker	absolut	^\s*är\s+inte\b
vet inte	betyder	bra fråga
övertygad inte	definitivt	chansa
inte övertygad	exakt	eventuellt
	helt klart	fattar inte
	helt säkert	förklara

Certainty disqualifiers	Certainty qualifiers	Uncertainty qualifiers
	innebär	försöka
	konstatera	förstår \w+ inte
	övertygad	förstår inte
	precis	frågan är
	säker	funderar
	säkert	gissa
	självklart	gissar
	tveklöst	glömt
	uppenbart	hjärn stopp
	vet	hur
		hur kommer
		hur får vi
		hur gör
		hur ska
		hur \w ska
		hur \w tycker
		ingenting jag ser
		inte \w+ säker
		inte ihåg
		inte säker
		kan inte
		kan nog
		kan vi \w+ veta
		kanske
		minns inte
		osäker
		ser du\??
		ser inte
		skulle kunna
		troligtvis
		tror
		tvekar
		tveksam
		vad gör vi
		vad ska jag
		vad skulle du göra\??
		vad tycker du\??
		vägleda
		väl
		vet \w+ inte
		vet inte

S4. Annotation codebook

Rule	Unit	If	Label / action	Notes
VC0	Eligibility	Speaker does not start with Subject	Ignore	Only student utterances are coded.
VC1	Unit definition	Row is a student utterance	Code one utterance	One transcript row is one coding unit.
VC2	Frozen cue lists	Coder/program is deciding stance	Use only cue lists	No new cues are added during coding or analysis.

Rule	Unit	If	Label / action	Notes
VC3	Uncertainty cue present	At least one uncertainty cue matches	Uncertain	Lexical or grammatical cues only; no pauses, correctness, or emotion.
VC4	Certainty cue present	At least one certainty cue remains after disqualifier removal	Certain	Disqualifiers are removed before certainty search.
VC5	Disqualifier blocking	Certainty term occurs only inside disqualifier phrase	Do not label certain	Prevents false hits such as vet inside vet inte.
VC6	Mixed-cue rule	Certain and uncertain cues occur in same utterance	Uncertain	Conservative rule: uncertainty overrides boosting.
VC7	hur constraint	Standalone hur appears	Count only as question-use	Counts only when used as a question word or in explicit multiword patterns.
VC8	precis constraint	precis appears	Count only as booster-use	Neutral when used only as assent/backchannel.
VC9	Evidence recording	Utterance is certain or uncertain	Record matched cues	Record exact matched cue substrings for auditability.
VC10	Per-utterance score	Label assigned	+1, -1, or 0	CERTAIN=+1, UNCERTAIN=-1, NEUTRAL=0.
VC11	Neutral default	No valid cue remains	Neutral	Agreement, answers, equations, theorem labels, and backchannels are neutral unless a cue matches.
VC12	Extraction row	Label is certain or uncertain	Output row	Extracted data contain non-neutral utterances with ID, label, and matched cues.

S5. Worked coding examples

The examples below illustrate how the rulebook was applied. They are short publication-facing examples based on the final coding logic; cue strings are shown in Swedish because the interaction was conducted in Swedish.

Case	Utterance / pattern	Cue	Label	Score	Rationale
Certainty qualifier	vi vet att	vet	CERTAIN	+1	The utterance contains an explicit knowledge marker.
Uncertainty qualifier	kanske	kanske	UNCERTAIN	-1	The utterance contains a hedge.
Disqualifier blocking	jag vet inte	vet inte	UNCERTAIN	-1	The phrase blocks vet from being counted as certainty.
Question-use constraint	Hur ska vi göra?	hur ska	UNCERTAIN	-1	hur is used as a task-related question word.
Neutral default	X är 16		NEUTRAL	0	A mathematical answer is neutral unless an epistemic cue appears.
Backchannel constraint	precis.	precis	NEUTRAL	0	precis used only as acknowledgement is not treated as certainty.
Mixed-cue rule	säkert, men jag tror...	säkert; tror	UNCERTAIN	-1	Uncertainty overrides boosting when both cue types occur.

S6. Annotation procedure and adjudication

The annotation used five inputs: transcripts, the frozen certainty lexicon, the program match list with provisional scores, Rater 1's verified output, and Rater 2's verified output. Reliability was evaluated at the utterance-label level. All human-rater disagreements were resolved by consensus before final scores were computed.

Human-rater disagreements were classified into the following types:

Mismatch type	Definition	All	Pre	Post
LEXICON_MATCH	Disagreement about whether a flagged phrase counts as an epistemic marker.	1	1	0
CONTEXT_SCOPE	Phrase is a marker but refers to non-task uncertainty.	1	1	0
MISS_ADD	One rater adds a non-neutral phrase missed by the other rater.	4	3	1
TRANSCRIPT_CUTOFF	A transcript-line boundary reduces local context for a cue.	1	1	0
CHALLENGE_CONTEXT	A cue can be interpreted as neutral when contesting a robot challenge.	4	1	3
WINDOW_BOUNDARY	Disagreement about whether phrase belongs to pre- or post-dissent window.	0	0	0
TOTAL	Total human-rater mismatches.	11	7	4

The most common disagreement types were missed additions and challenge-context cases. Challenge-context cases occurred when an uncertainty-like expression could be interpreted as part of contesting the robot's claim rather than as a direct marker of the student's own uncertainty.

S7. Reliability summary

The reliability set contained 207 utterance-level cases. The table reports agreement between the two human raters and between the deterministic program labels and the adjudicated human gold labels.

Window	Pair	N	Agreement (%)	Kappa
ALL	R1–R2	207	94.69	.895
ALL	Python–Gold	207	91.79	.828
PRE-DISSENT	R1–R2	173	95.95	.916
PRE-DISSENT	Python–Gold	173	94.22	.874
POST-DISSENT	R1–R2	34	88.24	.805
POST-DISSENT	Python–Gold	34	79.41	.634

Human-human agreement was high overall (94.69% agreement, $\kappa = .895$). Agreement remained high when pre-dissent and post-dissent windows were examined separately, although the post-dissent set was smaller and more difficult. Program-gold agreement was also high overall (91.79%, $\kappa = .828$), with lower agreement post-dissent, where short, challenge-bound responses were more context-sensitive.

S8. Interpretation limits

Verbal Certainty should be interpreted as a lexical interactional indicator, not as a direct measure of private belief, mathematical competence, or correctness. Four constraints are important:

- The score captures expressed certainty or uncertainty in speech, not unspoken confidence.
- The score is lexical and rule-bound: pauses, tone, gaze, facial expression, and correctness were not used in the label assignment.
- Utterances that only provided an answer, theorem name, equation, agreement token, or backchannel were neutral unless an explicit cue matched.
- Post-dissent labels were more context-sensitive because some uncertainty-like phrases occurred while students were actively contesting the robot's challenge.

Document note. This supplement was prepared as a publication-facing rulebook from the project workbook sheets on verbal certainty cue lists, annotation rules, adjudication, and reliability. Minor wording was standardised for readability; numeric values and coding rules follow the final analysis tables.
