# Supplementary material for: Persuasion, pride and prejudice: factors associated with students’ resistance to a social robot’s incorrect mathematical solution
Source: Front Robot AI. 2026 Sep 10;13:1901093. doi: 10.3389/frobt.2026.1901093 (PMC13601342; doi:10.3389/frobt.2026.1901093)
Supplement: Supplementary file 2 [file Supplementaryfile5.pdf]

Supplementary Material 5
Gaze annotation protocol and reliability

Associated manuscript: *Persuasion, Pride and Prejudice: Factors associated with students' resistance to a social robot's incorrect mathematical solution*.
Purpose: This supplement documents the gaze coding categories, annotation procedure, agreement checking, and interpretation limits used to construct the gaze variables reported in the manuscript. It is separate from Supplementary Material 4, which documents Verbal Certainty.

S1. Scope of this supplement

Gaze direction is an operational interactional measure of the student's visual orientation during the interaction. It captures whether the student was oriented toward the task, the robot, or elsewhere. It does not measure private attention directly, and it does not evaluate the student's reasoning, uncertainty, or confidence.

The coding procedure used manual annotation from video recordings, with the corrected transcript used as a synchronized time reference. Final analysis values were computed from consensus annotations after agreement checking on a randomly selected subset of participants.

S2. Unit of analysis and gaze categories

Element	Operational definition
Coded unit	One gaze state, beginning at an annotated gaze-shift timestamp and remaining active until the next annotated shift.
Speaker eligibility	Only student gaze was coded. Robot gaze and experimenter gaze were ignored.
Starting state	At timestamp 0, the student's initial gaze category was recorded.
Subsequent coding	Only gaze shifts were annotated. Each new timestamp marked a change from the current category to a new category.
Category set	TASK, ROBOT, or AWAY.
Comparison unit	For agreement checking, gaze streams were reconstructed into 125-ms segments.
Aggregation	Segment durations were summed within participant-phase windows and converted to gaze-time proportions.

The gaze categories were mutually exclusive. *Task* indicated gaze toward the task sheet or task-relevant written material on the table. *Robot* indicated gaze toward the robot. *Away* indicated gaze elsewhere, including off-task locations, experimenters, or indeterminate non-task/non-robot locations.

S3. Annotation procedure

Annotations were produced from the video recordings using the corrected Sonix.ai transcript as a synchronized time reference. At timestamp 0, annotators recorded the student's initial gaze category. After that point, only gaze shifts were annotated. When the student shifted gaze away from the current category, the annotator paused the video at the estimated shift point and entered the timestamp together with the new gaze category in the transcript.

The resulting annotation represented a sequence of gaze states rather than frame-by-frame coding. Each category remained active until the next annotated shift. Ambiguous cases were handled by forced choice among *task*, *robot*, and *away*; annotators were instructed not to create additional uncertainty categories.

S4. Annotation codebook

Rule	Unit	If	Label / action	Notes
GZ0	Eligibility	The visible person is not the student	Ignore	Only student gaze is coded.
GZ1	Initial state	Timestamp is 0	Record current gaze category	The first category starts the gaze-state sequence.
GZ2	Shift coding	Student shifts gaze away from current category	Record timestamp and new category	No annotation is entered if gaze remains in the current category.
GZ3	Task gaze	Student looks toward the task sheet or task-relevant written material	TASK	This is the focal gaze category in the main analyses.
GZ4	Robot gaze	Student looks toward the robot	ROBOT	Includes gaze directed to the robot face/head.
GZ5	Away gaze	Student looks elsewhere	AWAY	Includes off-task locations, experimenters, or indeterminate non-task/non-robot locations.
GZ6	Forced-choice rule	Gaze shift is visible but target is ambiguous	Choose TASK, ROBOT, or AWAY	No additional uncertainty category is used.
GZ7	State persistence	No later shift is annotated	Keep previous category active	Each category remains active until the next shift.
GZ8	Agreement reconstruction	Comparing two annotations	Reconstruct into 125-ms segments	Each segment inherits the category active at that time.
GZ9	Discrepancy review	Rater streams disagree after reconstruction	Inspect and resolve by consensus	Final analysis uses consensus annotations.

S5. Worked coding examples

The examples below illustrate how the rulebook was applied. They are publication-facing examples based on the final coding logic.

Case	Observed pattern	Label / action	Rationale
Initial task gaze	At timestamp 0, the student is looking at the task sheet.	TASK	The initial gaze state is recorded before shift coding begins.
Shift to robot	The student lifts their gaze from the task sheet to the robot.	Timestamp + ROBOT	The timestamp marks the start of a new gaze state.
Shift back to task	The student looks from the robot back to the task sheet.	Timestamp + TASK	The previous ROBOT state remains active until this shift.
Look elsewhere	The student turns toward an experimenter or another location in the room.	Timestamp + AWAY	The gaze is neither task-directed nor robot-directed.
Ambiguous robot/away case	The student turns their head near the robot direction, but the exact target is unclear.	Forced choice	The annotator selects the most likely of TASK, ROBOT, or AWAY according to the video evidence.
No shift	The student continues looking at the task sheet while speaking.	No new row	Gaze states are not re-entered unless a shift occurs.

S6. Agreement checking and adjudication

A second annotator independently checked the full gaze annotations for six randomly selected participants, corresponding to 27.3% of the sample. To compare the two annotations, a script reconstructed each gaze stream into 125-ms segments. For each segment, the active gaze category was inferred from the most recent annotated gaze shift. This produced directly comparable time-aligned category sequences for the two annotators.

All remaining discrepancies identified in the checked subset were inspected by the researchers. Disagreements about gaze category or segment boundary were resolved by consensus before final gaze variables were computed.

S7. Reliability summary

The agreement set contained 18,092 reconstructed 125-ms segments from six randomly selected participants. The table reports non-matching segments by category pair and the corresponding segment-level agreement rates.

Participant	Segments	R–A	R–T	T–A	R–A rate	R–T rate	T–A rate	Total rate
1	4140	156	0	66	.9623	1.0000	.9841	.9464
6	2372	46	10	3	.9806	.9958	.9987	.9751
11	2491	86	2	25	.9655	.9992	.9900	.9546
2	3713	42	3	12	.9887	.9992	.9968	.9846
15	3101	43	9	3	.9861	.9971	.9990	.9823
8	2275	12	2	0	.9947	.9991	1.0000	.9938
TOTAL	18092	385	26	109	.9787	.9986	.9940	.9713

R–A = robot–away discrepancy; R–T = robot–task discrepancy; T–A = task–away discrepancy. The overall segment-level agreement was 97.13%. Most non-matching segments involved the distinction between *robot* and *away*. Disagreements involving *task*, the focal gaze category in the main analyses, were rare: R–T and T–A discrepancies together accounted for 135 of 18,092 segments.

S8. Interpretation limits

Gaze variables should be interpreted as video-based indicators of visual orientation, not as eye-tracking measures. Four constraints are important:

- The score captures visible gaze orientation from the camera perspective, not private attention.
- The score is based on manual video annotation and timestamped gaze shifts; exact shift boundaries are approximate.
- Camera angle and participant head orientation sometimes made fine distinctions between *robot* and *away* more difficult.
- The most robust gaze distinction was between *task* and non-task gaze; accordingly, *Gaze Time at Task* was treated as the focal gaze variable in the manuscript.

Document note. This supplement was prepared as a publication-facing rulebook from the project gaze annotation protocol, rater-comparison script output, and agreement summary. Minor wording was standardised for readability; numeric values and coding rules follow the final analysis tables.
