# Supplementary material for: Persuasion, pride and prejudice: factors associated with students’ resistance to a social robot’s incorrect mathematical solution
Source: Front Robot AI. 2026 Sep 10;13:1901093. doi: 10.3389/frobt.2026.1901093 (PMC13601342; doi:10.3389/frobt.2026.1901093)
Supplement: Supplementary file 3 [file Supplementaryfile6.pdf]

Förhandsgranskningsläge

Publicerat

Kopiera svarslänk

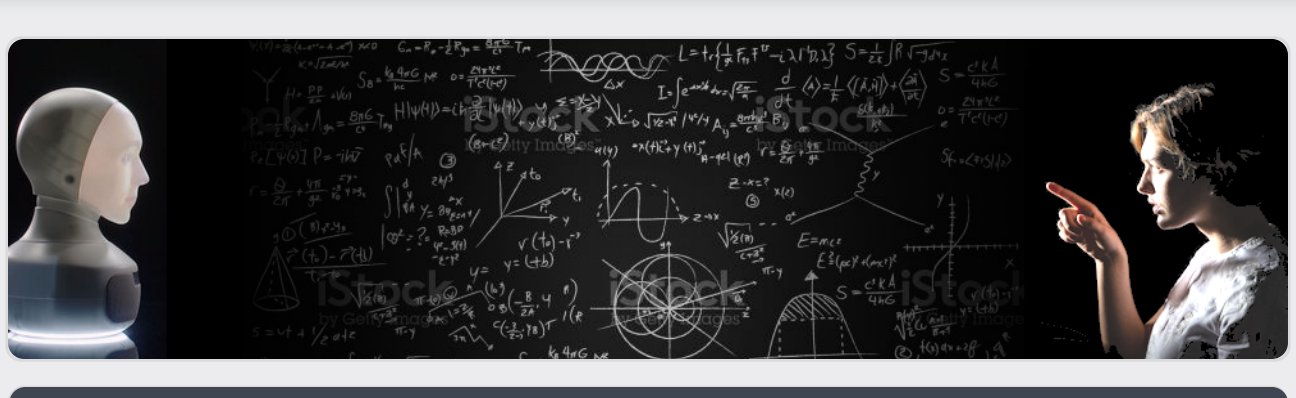

Pre-session Survey

Gender

- ◯ Female
- ◯ Male
- ◯ Non-binary
- ◯ Prefer not to say
- ◯ Other: ______

Age:

Välj ▾

Country of Origin:

- ◯ Sweden
- ◯ Other: ______

What do you consider to be your native language?

- ◯ Swedish
- ◯ Other: ______

I am really good at Math

- ◯ Strongly disagree
- ◯ Disagree to some extent
- ◯ Neutral
- ◯ Agree to some extent
- ◯ Strongly agree

I do enjoy Math

- ◯ Strongly disagree
- ◯ Disagree to some extent
- ◯ Neutral
- ◯ Agree to some extent
- ◯ Strongly agree

How would you rank the following topics based on your level of enjoyment, from the most enjoyable to the least enjoyable?

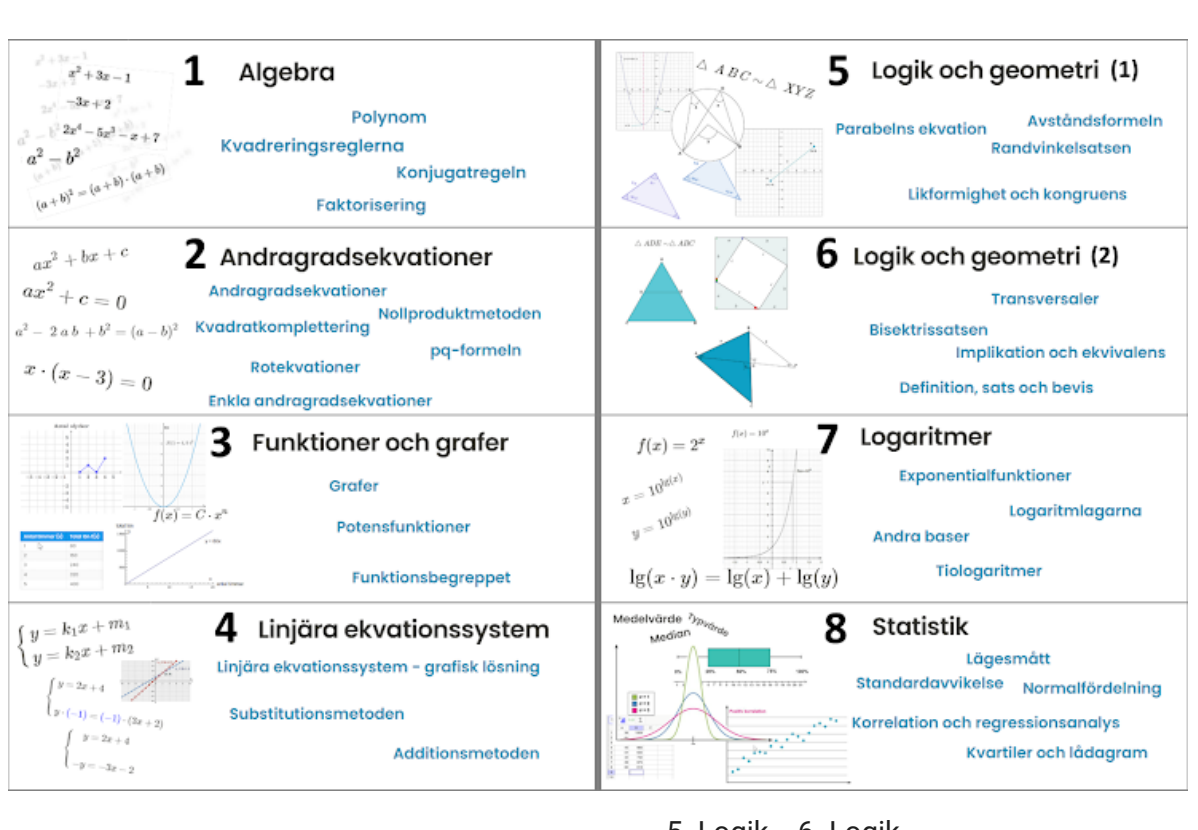

	1. Algebra	2. Andra grads ekvationer	3. Funk tioner	4. Linjära ekvations system	5. Logik och geometri [1]	6. Logik och geometri [2]	6. Logaritmer	7. Statistik
1st	◯	◯	◯	◯	◯	◯	◯	◯
2nd	◯	◯	◯	◯	◯	◯	◯	◯
3rd	◯	◯	◯	◯	◯	◯	◯	◯
4th	◯	◯	◯	◯	◯	◯	◯	◯
5th	◯	◯	◯	◯	◯	◯	◯	◯
6th	◯	◯	◯	◯	◯	◯	◯	◯
7th	◯	◯	◯	◯	◯	◯	◯	◯
8th	◯	◯	◯	◯	◯	◯	◯	◯

For each topic, how do you think you would perform in an exam?

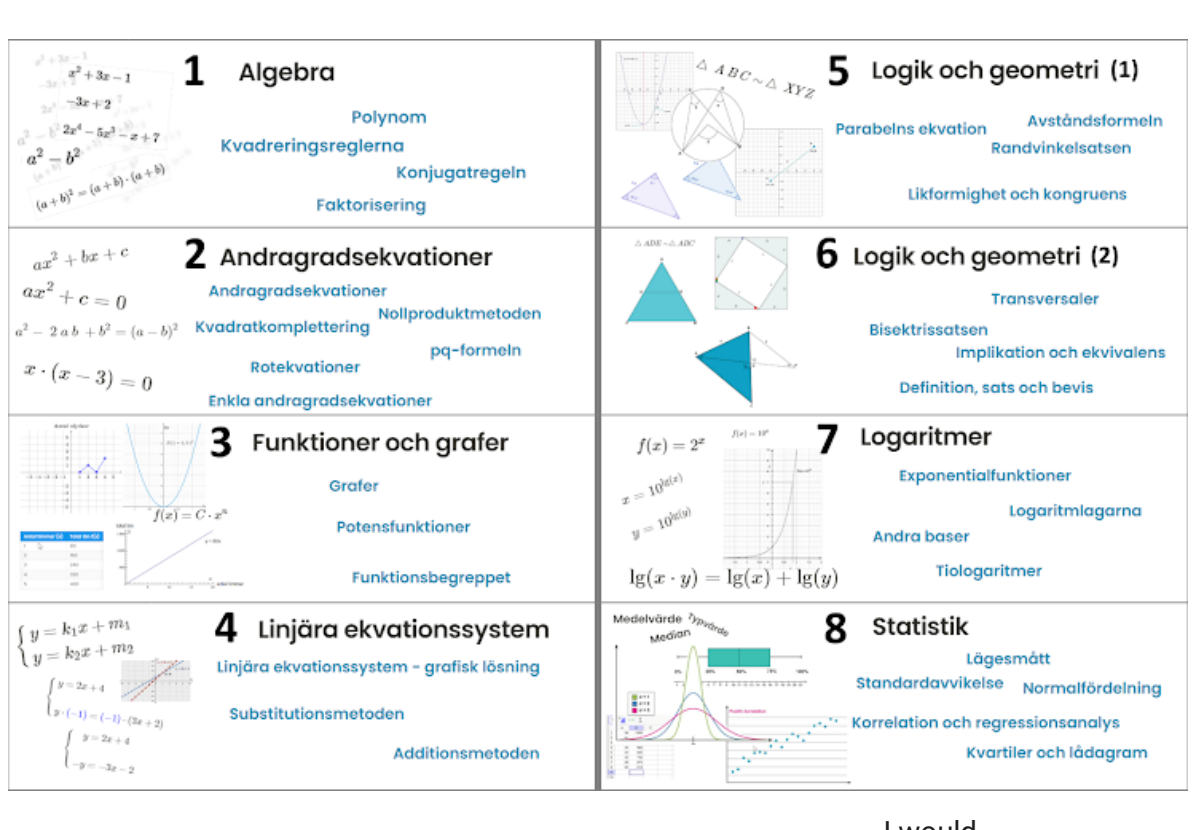

	I would fail 100%	I would probably fail	I don't know	I would probably pass	I would pass 100%
Algebra	◯	◯	◯	◯	◯
Andragradsekvationer	◯	◯	◯	◯	◯
Funktioner och grafer	◯	◯	◯	◯	◯
Linjära ekvationssystem	◯	◯	◯	◯	◯
Logik och geometri (1)	◯	◯	◯	◯	◯
Logik och geometri (2)	◯	◯	◯	◯	◯
Logaritmer	◯	◯	◯	◯	◯
Statistik	◯	◯	◯	◯	◯

I always follow advice from my professors.

- ◯ Strongly disagree
- ◯ Disagree to some extent
- ◯ Neutral
- ◯ Agree to some extent
- ◯ Strongly agree

When a professor tells me something I tend to believe it is true.

- ◯ Strongly disagree
- ◯ Disagree to some extent
- ◯ Neutral
- ◯ Agree to some extent
- ◯ Strongly agree

Whenever I commit to an appointment I do as I told.

- ◯ Strongly disagree
- ◯ Disagree to some extent
- ◯ Neutral
- ◯ Agree to some extent
- ◯ Strongly agree

I try to do everything I have promised to do.

- ◯ Strongly disagree
- ◯ Disagree to some extent
- ◯ Neutral
- ◯ Agree to some extent
- ◯ Strongly agree

If someone from my social network notifies me about something to consult, I tend to do it.

- ◯ Strongly disagree
- ◯ Disagree to some extent
- ◯ Neutral
- ◯ Agree to some extent
- ◯ Strongly agree

When I am in a new situation I look at others to see what I should do.

- ◯ Strongly disagree
- ◯ Disagree to some extent
- ◯ Neutral
- ◯ Agree to some extent
- ◯ Strongly agree

I accept advice from my social network.

- ◯ Strongly disagree
- ◯ Disagree to some extent
- ◯ Neutral
- ◯ Agree to some extent
- ◯ Strongly agree

When I like someone, I am more inclined to believe him or her.

- ◯ Strongly disagree
- ◯ Disagree to some extent
- ◯ Neutral
- ◯ Agree to some extent
- ◯ Strongly agree

I am using the following voice assistants / AI systems on a regular basis (several choices possible)

- ☐ Apple Siri
- ☐ Google Assistant
- ☐ Google Home
- ☐ Amazon Alexa
- ☐ Open AI (GPT3/GPT4)
- ☐ None
- ☐ Övrigt: ______

Describe your previous experience of interacting with a speaking robot

- ◯ None
- ◯ Scarce
- ◯ Some
- ◯ Regular
- ◯ Expert

Describe your general attitude towards interacting with a speaking robot

- ◯ Very uneasy
- ◯ Somewhat uneasy
- ◯ Neutral
- ◯ Mostly relaxed
- ◯ Very relaxed

In my opinion, robots should be developed to be as human-like as possible when it comes to APPEARANCE

- ◯ Strongly disagree
- ◯ Disagree
- ◯ Neutral
- ◯ Agree
- ◯ Strongly Agree

In my opinion, robots should be developed to be as human-like as possible when it comes to INTERACTION STYLE

- ◯ Strongly disagree
- ◯ Disagree
- ◯ Neutral
- ◯ Agree
- ◯ Strongly Agree

In my opinion, robots should be developed to be as human-like as possible when it comes to its VOICE AND SPOKEN UTTERANCES

- ◯ Strongly disagree
- ◯ Disagree
- ◯ Neutral
- ◯ Agree
- ◯ Strongly Agree

In my opinion, robots should be developed to be as human-like as possible when it comes to UNDERSTANDING SPEECH

- ◯ Strongly disagree
- ◯ Disagree
- ◯ Neutral
- ◯ Agree
- ◯ Strongly Agree

In my opinion, robots should be developed to be as human-like as possible when it comes to EMOTIONS

- ◯ Strongly disagree
- ◯ Disagree
- ◯ Neutral
- ◯ Agree
- ◯ Strongly Agree

I think that it would be a good idea to employ robots (now or in the future) in (choose all you agree with)

- ☐ Education
- ☐ Entertainment (e.g., social interaction)
- ☐ Service sector (e.g., providing information)
- ☐ Domestic (e.g., cleaning)
- ☐ Healthcare (e.g., social companion or screening of patients)
- ☐ Medicine (e.g., surgery)
- ☐ Industry
- ☐ Military/Security
- ☐ None of the above

Skicka

Rensa formuläret

Skicka aldrig lösenord med Google Formulär

Det här innehållet har varken skapats eller godkänts av Google. - Ägare av kontaktformulär - Användarvillkor - Integritetspolicy

Ser det här formuläret misstänkt ut? Rapport

Google Formulär

Förhandsgranskningsläge

Publicerat

Kopiera svarslänk

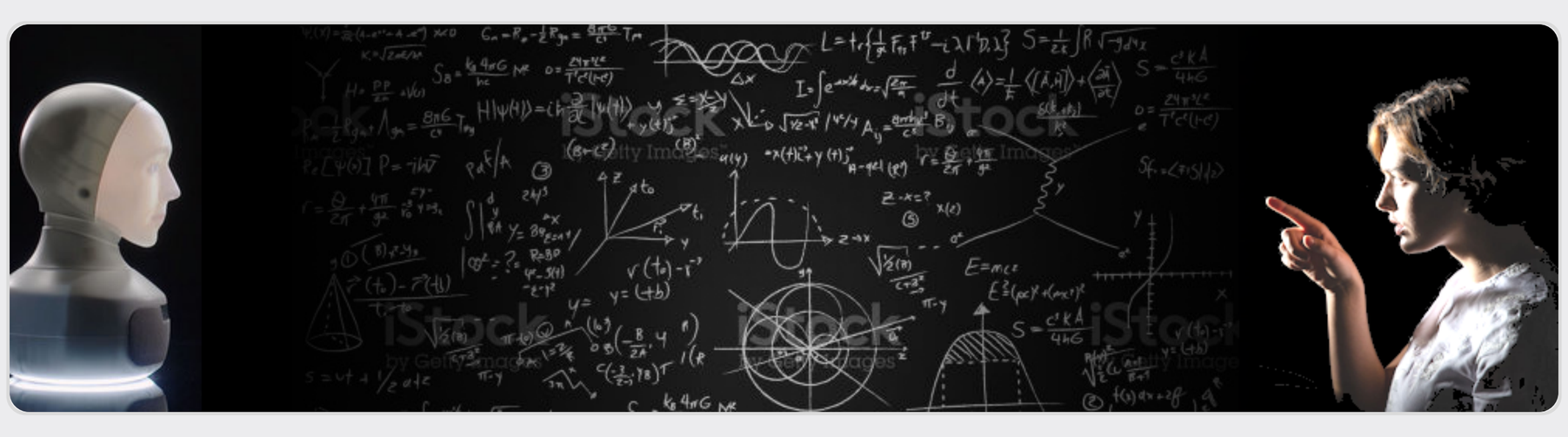

Post-session survey

How much do you trust the robot as a partner for this type of maths exercise?

	0	1	2	3	4	5	
Nothing	○	○	○	○	○	○	Completely

During the activity, you discussed the triangle (Stage 1), the angle a (Stage 2) and the circle (Stage 3).

Did you both have some INITIAL disagreement in any of the stages?

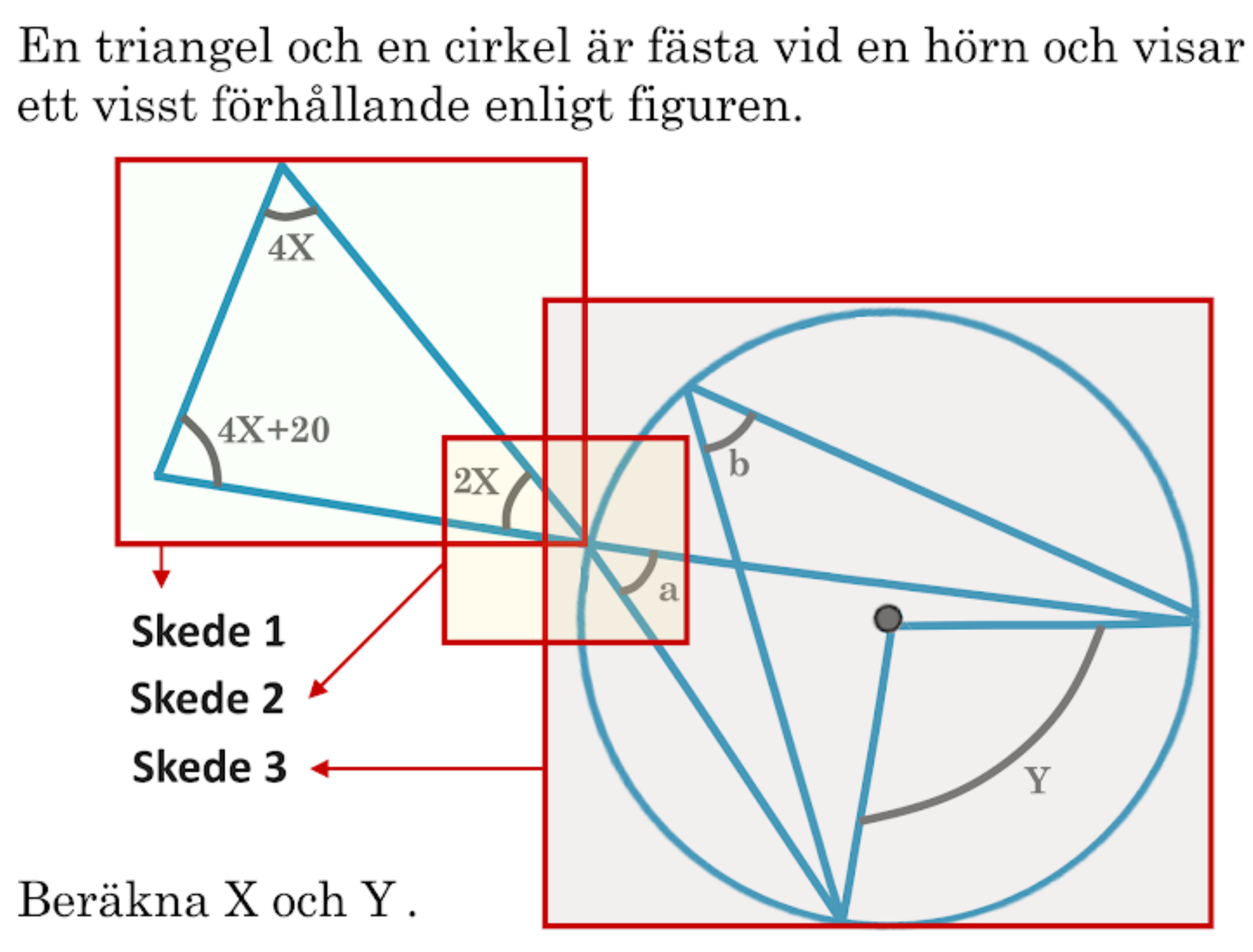

- ○ Yes, at Stage 1
- ○ Yes, at Stage 2
- ○ Yes, at Stage 3
- ○ No disagreement
- ○ Övrigt: ______

How did the disagreement get resolved?

- ○ The robot convinced me of its solution
- ○ I convinced the robot of my solution

[ACTIVATED IF ANSWER TO PREVIOUS QUESTION WAS The robot convinced me of its solution] Before the robot convinced you, what was your position when you started the discussion?

- ○ I was blank. I didn't know anything about it.
- ○ I thought the same as the robot, but I was not sure.
- ○ I was doubting between the robot's position and another one.
- ○ I thought something different than the robot's position, but was not sure enough.
- ○ I was convinced about something different than the robot's position.
- ○ Övrigt: ______

[ACTIVATED IF ANSWER TO PREVIOUS QUESTION WAS I convinced the robot of my solution]
Before you convinced the robot, what was your position when you started the discussion?

- ○ Initially I didn't know anything about it, and then remembered.
- ○ Initially I thought the same as the robot, but then changed my mind.
- ○ Initially I was doubting between the robot's position and mine.
- ○ Initially I thought the same as now, but less convinced.
- ○ Initially I thought the same as now and equally convinced.
- ○ Initially I thought the same as now but more convinced.
- ○ Övrigt: ______

How correct did *the robot's solution* seem to you when **you started the discussion**?

	1	2	3	4	5	
Completely incorrect	○	○	○	○	○	Completely correct

How correct does *the robot's solution* seem to you **now**?

	1	2	3	4	5	
Completely incorrect	○	○	○	○	○	Completely correct

How do you think that the robot perceived your position when **you started the discussion**?

	1	2	3	4	5	
Completely incorrect	○	○	○	○	○	Completely correct

How do you think that the robot perceives your solution **now**?

	1	2	3	4	5	
Completely incorrect	○	○	○	○	○	Completely correct

The robot influenced my thinking (regardless of if it was right or wrong).

- ○ Disagree completely
- ○ Disagree to some extent
- ○ Neutral
- ○ Agree to some extent
- ○ Agree completely

The robot made me change how I was thinking (regardless of if it was right or wrong).

- ○ Disagree completely
- ○ Disagree to some extent
- ○ Neutral
- ○ Agree to some extent
- ○ Agree completely

The robot was convincing (regardless of if it was right or wrong).

- ○ Disagree completely
- ○ Disagree to some extent
- ○ Neutral
- ○ Agree to some extent
- ○ Agree completely

The robot seemed reliable

- ○ Disagree completely
- ○ Disagree to some extent
- ○ Neutral
- ○ Agree to some extent
- ○ Agree completely

How would you rate the session from a learning perspective?

	0	1	2	3	4	5	
Poor	○	○	○	○	○	○	Excellent

Other comments about the session

Ditt svar

Skicka

Sidan 1 av 1

Rensa formuläret

Skicka aldrig lösenord med Google Formulär

Det här innehållet har varken skapats eller godkänts av Google. - Ägare av kontaktformulär - Användarvillkor - Integritetspolicy

Ser det här formuläret misstänkt ut? Rapport

Google Formulär
